# Comparative analysis of medicinal plants *Scutellaria baicalensis* and common adulterants based on chloroplast genome sequencing

**DOI:** 10.1186/s12864-023-09920-2

**Published:** 2024-01-08

**Authors:** Zhen Li, Baozhong Duan, Zhongyu Zhou, Hui Fang, Meihua Yang, Conglong Xia, Ying Zhou, Jing Wang

**Affiliations:** 1 College of Pharmaceutical Science, Dali University, Dali, 671000 China; 2 Institute of Caulis Dendrobii of Longling County, Baoshan, 678300 China; 3 College of Life Science, Northeast Forestry University, Harbin, 150040 China; 4 Key Laboratory of Natural Medicines of the Changbai Mountain, Ministry of Education, Yanbian University, Yanji, 133002 China

**Keywords:** *Scutellaria baicalensis*, Chloroplast genome, Species identification, Phylogenetic, *Scutellaria*

## Abstract

**Background:**

*Scutellaria baicalensis* Georgi has been extensively used as a medicinal herb in China for over 2000 years. They may be intentionally or inadvertently substituted or blended with comparable species in the local market, threatening clinical medication safety. Molecular markers are effective tools to prevent misidentification and eliminate doping and falsification among *Scutellaria* plants. This study screened four highly variable regions to identify *Scutellaria* and its adulterants. In addition, a phylogenetic analysis was performed using the complete cp genome combined with published *Scutellaria* species samples. Moreover, a comparative analysis of the cp genomes was conducted to investigate the cp genome evolution of *S. baicalensis*.

**Results:**

The complete cp genome of five species of *Scutellaria* was sequenced for the first time, and four previously published *Scutellaria* species were re-sequenced. They all exhibited a conserved quadripartite structure in their cp genomes, including two distinct regions, namely a small and large single copy region, respectively, and two inverted repeats encompassing the majority of ribosomal RNA genes. Furthermore, the nine species exhibited high conservation from aspects of the genome structure, codon usage, repeat sequences, and gene content. Four highly variable regions (*matK-rps16*, *ndhC-trnV-UAC*, *psbE-petL*, and *rps16-trnQ-UUG*) may function as potential molecular markers for differentiating *S. baicalensis* from its adulterants. Additionally, the monophyly of *Scutellaria* was ascertained and could be reclassified into two subgenera, subgenus *Anaspis* and subgenus *Scutellaria*, as evidenced by the phylogenetic analyses on sequences of cp genome and shared protein-coding sequences. According to the molecular clock analysis, it has been inferred that the divergence of *Scutellaria* occurred at approximately 4.0 Mya during the Pliocene Epoch.

**Conclusion:**

Our study provides an invaluable theoretical basis for further *Scutellaria* species identification, phylogenetics, and evolution analysis.

**Supplementary Information:**

The online version contains supplementary material available at 10.1186/s12864-023-09920-2.

## Background

*Scutellaria baicalensis* Georgi, a member of the Lamiaceae family and commonly known as "Huang qin" in Chinese, has been listed in the Chinese Pharmacopoeia due to its critical medicinal properties, such as clearing heat, purging fire, eliminating dampness, preventing miscarriage, and maintaining hemostasis [1]. However, previous studies showed that *S. baicalensis* is often contaminated by other common adulterants, such as *S. indica* L., *S. yunnanensis* H.Lév., *S. tenax* W.W.Sm., *S. forrestii* Diels, and *S. caryopteroides* Hand.-Mazz [2]*.* These substitute herbs are commonly of substandard quality and may even contain harmful constituents [3]. Given their similar morphology and shared vernacular name, distinguishing them solely by sight can be highly challenging and misleading. Currently, the use of molecular markers has made noteworthy advancements in the field of Chinese medicine identification. This approach aims to discriminate individuals from populations by sequencing particular genomic regions [4]. The utilization of universal DNA barcodes, specifically *matK*, *rpl32-trnl*, *ndhF-rpl32*, and *trnL-trnF*, have been employed to differentiate and verify *S. baicalensis* from its dopants [5–8]. However, these investigations did not cover certain prevalent adulterants, and single-locus DNA barcodes have inherent limitations [2, 9]. Subsequent experiments have identified shortcomings in the universal DNA barcodes for identifying common contaminants in the market, as illustrated in Fig. S1 and Fig. S2. Therefore, it is crucial to develop a more accurate and effective method for distinguishing *S. baicalensis* from its common contaminants.

The chloroplast (cp) is involved in plant photosynthesis and numerous biochemical processes [10, 11]. Unlike the nuclear genome, the cp genome is comparatively stable, exhibiting only minor variations [12, 13], which has been successfully employed for discriminating *Amomum, Isodon,* and their contaminants [14, 15]. Although the cp genomes of some *Scutellaria* species have been described [16, 17], these studies mainly characterized a single genome or compared the intraspecific variation. Nonetheless, *Scutellaria* species and their common adulterants have never been discriminated by comparing their cp genomes. In addition, gene-encoding regions diverge more slowly than non-coding regions, and therefore, they could offer an improved resolution for understanding phylogenetic relationships [18, 19]. Nevertheless, protein-coding genes have not been used to comprehend the evolutionary relationship of *Scutellaria*.

Here, *de novo* sequencing and assembly, as well as annotation of the cp genomes of nine distinct *Scutellaria* species, were conducted to (i) enhance the comprehension of the overall cp genome structure of *Scutellaria*, (ii) elucidate the phylogenetic relationships of *Scutellaria*, and (iii) filter candidate molecular markers to differentiate *S. baicalensis* from its adulterants. Therein, the cp genomes of five related species, including *S. indica*, *S. yunnanensis*, *S. tenax*, *S. forrestii*, and *S. caryopteroides*, were published for the first time. The findings enrich the available genomic resources for *Scutellaria* by offering crucial information to support phylogenetic analysis, distinguish the *Scutellaria* genus, and facilitate the safe medical applications of *S. baicalensis*.

## Results

### Characterization of cp genomes

**Fig. 1:**
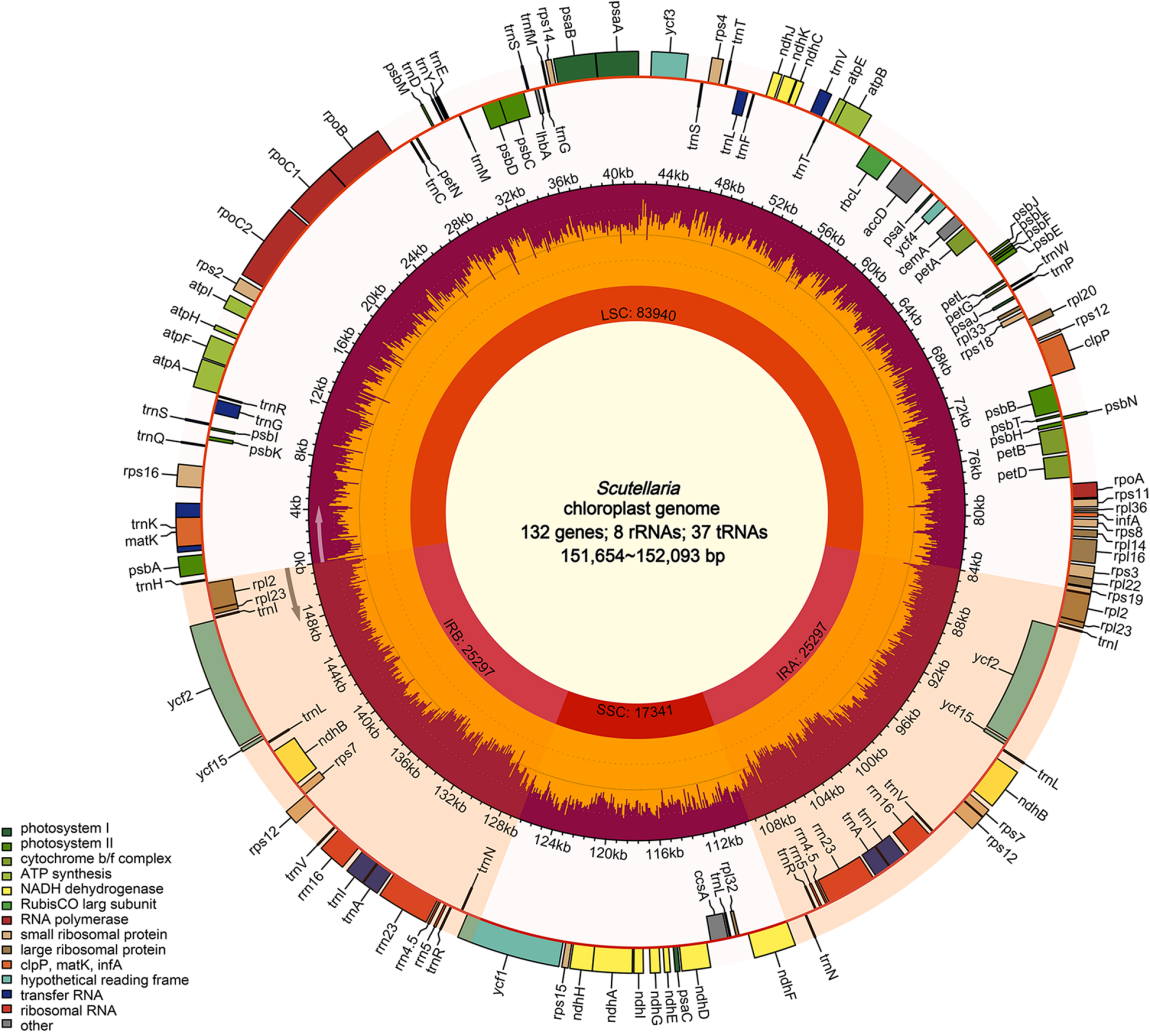
Cp genome map of *Scutellaria*. Genes inside the circle are transcribed clockwise, while those outside are transcribed counterclockwise

For each species, clean data totaling approximately 2-5 Gb were acquired. The complete cp genomes were obtained after assembly and splicing (Fig. 1). The annotated results showed that among the nine species, the cp genome of *S. likiangensis* was the smallest, with 151,654 bp, while that of S. *tenax* was the largest (152,093 bp) (Table 1). The length of the LSC, SSC, and IR regions varied from 83,741 bp (*S. forrestii*) to 84,102 bp (*S. caryopteroides*), 17,326 bp (*S. likiangensis*) to 17,549 bp (*S. tenax*), and 25,229 bp (*S. caryopteroides*) to 25,255 bp (*S. amoena*), respectively.

**Table 1:** Summary of cp genome features

**Species**	**Total length (bp)**	**GC content (%)**	**AT content (%)**	**LSC length (bp)**	**SSC length (bp)**	**IR length (bp)**	**Gene number**	**Protein-coding gene number**	**rRNA gene number**	**tRNA gene number**	**GenBank accession**
***S. likiangensis***	151654	38.4	61.6	83822	17326	25253	130	86	8	36	OP597811
***S. tenax***	152093	38.4	61.6	84058	17549	25243	132	87	8	37	OP597812
***S. barbata***	152091	38.3	61.7	84090	17531	25235	130	86	8	36	OP597813
***S. baicalensis***	151875	38.3	61.7	83940	17341	25297	132	87	8	37	OP597814
***S. yunnanensis***	152051	38.3	61.7	84050	17531	25235	132	87	8	37	OP597819
***S. indica***	152003	38.4	61.6	84003	17534	25233	124	85	8	31	OP597815
***S. caryopteroides***	152073	38.4	61.6	84102	17513	25229	130	86	8	36	OP597816
***S. forrestii***	151759	38.4	61.6	83741	17534	25242	130	86	8	36	OP597817
***S. amoena***	151840	38.4	61.6	83990	17340	25255	132	87	8	37	OP597818

From the cp genomes of nine species, 124-132 genes were identified, including 85-87 protein-coding genes, 31-37 tRNA genes, and eight rRNA genes. Therein, 18 genes have two replicates in IR regions (Table 1). Briefly, 12 repeat genes were located in the LSC, including *trnK-UUU*, *trnG-GCC*, *trnL-UAA*, *trnV-UAC*, *clpP*, *petB*, *petD*, *rpl16*, *rps16*, *atpF*, *rpoC1*, and *ycf3*. Four repetitive genes in the IR included *ndhB*, *rpl2*, *trnI-GAU*, and *trnA-UGC*, whereas *ndhA* was the only one present in the SSC region. Notably, *Scutellaria* contained 16 repeated genes involved in photosynthesis and self-replication (Table 2). Additionally, the cp genomes among the studied species displayed high conservation in the GC content at approximately 38.3%.

**Table 2:** List of genes in the cp genome of the nine *Scutellaria* species

**Gene function**	**Group of genes**	**Gene names**	**Amount**
rRNA	rRNA genes	*rrn16S*(×2), *rrn23S*(×2), *rrn4.5S*(×2), *rrn5S*(×2)	8
tRNA	tRNA genes	*trnH-GUG*, *trnK-UUU*, *trnQ-UUG*, *trnS-GCU*, *trnG-GCC*, *trnR-UCU*, *trnC-GCA*, *trnD-GUC*, *trnY-GUA*, *trnE-UUC*, *trnM-CAU*, *trnS-UGA*, *trnG-UCC*, *trnfM-CAU*, *trnS-GGA*, *trnT-UGU*, *trnL-UAA*, *trnF-GAA*, *trnV-UAC*, *trnT-GGU*, *trnW-CCA*, *trnP-UGG*, *trnI-CAU*(×2), *trnL-CAA*(×2), *trnV-GAC*(×2), *trnI-GAU*(×2), t*rnA-UGC*(×2), *trnR-ACG*(×2), *trnN-GUU*(×2), *trnL-UAG*	37
Self replication	Large subunit of ribosome	*rpl14*, *rpl16*, *rpl2*(×2), *rpl20*, *rpl22*, *rpl23*(×2), *rpl32*, *rpl33*,* rpl36*	11
	DNA dependent RNA polymerase	*rpoA*, *rpoB*, *rpoC1*,* rpoC2*	4
	Small subunit of ribosome	*rps11*, *rps12(*×2), *rps14*, *rps15*, *rps16*, *rps18*, *rps19*, *rps2*, *rps3*, *rps4*, *rps7*(×2),* rps8*	14
Photosynthesis	Subunits of ATP synthase	*atpA*, *atpB*, *atpE*, *atpF*, *atpH*, *atpI*	6
	Subunits of photosystem II	*lhbA*, *psbA*, *psbB*, *psbC*, *psbD*, *psbE*, *psbF*, *psbI*, *psbJ*, *psbK*, *psbL*, *psbM*, *psbN*, *psbT*,* ycf3*	15
	Subunits of NADH-dehydrogenase	*ndhA*, *ndhB*(×2), *ndhC*, *ndhD*, *ndhE*, *ndhF*, *ndhG*, *ndhH*, *ndhI*, *ndhJ*, *ndhK*	12
	Subunits of cytochrome b/f complex	*petA*, *petB*, *petD*, *petG*, *petL*, *petN*	6
	Subunits of photosystem I	*psaA*, *psaB*, *psaC*, *psaI*, *psaJ*	5
	Subunit of rubisco	*rbcL*	1
Other genes	Subunit of Acetyl-CoA-carboxylase	*accD*	1
	c-type cytochrom synthesis gene	*ccsA*	1
	Envelop membrane protein	*cemA*	1
	Protease	*clpP*	1
	Translational initiation factor	*infA*	1
	Maturase	*matK*	1
Unknown	Conserved open reading frames	*ycf1*, *ycf15*(×2), *ycf2*(×2), *ycf4*	6
Total			131

### Codon usage analysis

**Fig. 2:**
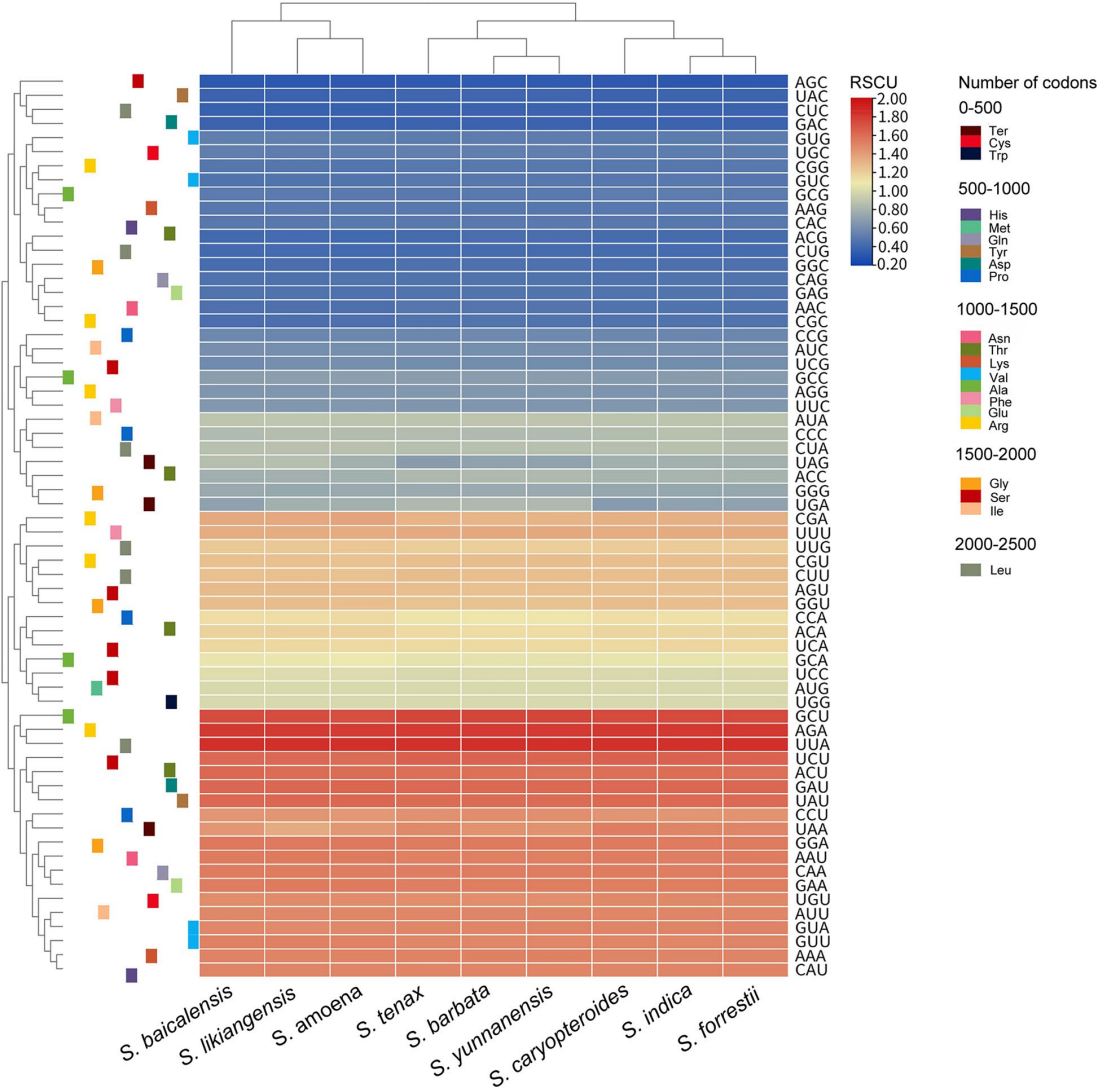
Heatmap of the RSCU values among nine *Scutellaria* species

The amino acid frequency, the number and bias of codon usage, and RSCU were investigated among the nine *Scutellaria* species cp genomes. The results indicated that 21 different amino acids were encoded in the cp genome, and a comprehensive set of 64 codons were deduced. Among these codons, 30 were frequently utilized in various *Scutellaria* species (Table S1-S9). 31 codons displayed RSCU values >1, 28 of which have A or U terminal nucleotides. The rest of the 33 codons had RSCU values ≤1, where 30 of these ended in G or C nucleotides. Additionally, the codon UUA had the highest frequency, followed by AGA, while AGC was the least common (Fig. 2). Leucine and cysteine had the highest and least number of codons, respectively. Moreover, unlike other amino acids were encoded by at least two synonymous codons, methionine, and tryptophan were encoded by only one respective codon. The GC content of synonymous third codon positions (GC3s) is correlated with codon bias to evaluate codon usage patterns. In nine species of *Scutellaria*, the GC3s values ranged from 38.20% to 39.10%, indicating a strong bias towards A/U-ending codons. Besides, both optimal frequency values and the codon adaptation index were less than 0.5, and the effective proportion of codons ranged from 55.47% to 55.90% (Table S10). The codon usage of cp genomes in *S. baicalensis*, *S. likiangensis*, and *S. amoena* was relatively similar (Fig. 2), suggesting that a minor bias existed in codon use across the nine *Scutellaria* species.

### Repeat analysis

Repetitive sequences have a significant impact on genome evolution and rearrangements. A total of 273 long repeats, including 144 P repeats, 128 F repeats, and one R repeat, were identified, while C repeats were absent (Fig. S3 A and Table S11). The repeats ranged in length from 30 bp to 129 bp, with 30 bp-39 bp being the most frequent and those longer than 70 bp being the least abundant (Table S11). Moreover, as the Hamming distance decreased from 3 to 1, the number of repeat sequences decreased significantly from 273 to 86 (Table S12).

SSRs are important genetic markers that facilitate the identification of closely related species [20, 21]. A total of 33,44, 34, 31, 44, 36, 33, 37, and 41 SSRs were obtained in the cp genomes of *S. likiangensis*, *S. tenax*, *S. barbata*, *S. baicalensis*, *S. yunnanensis*, *S. indica*, *S. caryopteroides*, *S. forrestii*, and *S. amoena*, respectively (Table S13, Fig. S3 B). In this study, the analysis of SSRs revealed that mononucleotide repeats in identified SSRs proportioned 54.55% - 64.86%, with the A/T motif being the most prevalent. The subsequent most commonly observed SSR types were dinucleotide repeats (12.20% - 18.18%) with a predominance of the AT/TA motif and tetranucleotide repeats (10.81% - 15.91%) with a predominance of the ATTT/AAAT motif. Trinucleotide repeats accounted for 3.22% - 9.76% of the SSRs dominated by the AAT/ATT motif. Finally, hexanucleotide repeats were observed at 0% - 16.13%, while pentanucleotide repeats were present at a frequency of 0% - 6.06%.

### Contraction and expansion of IRs

**Fig. 3:**
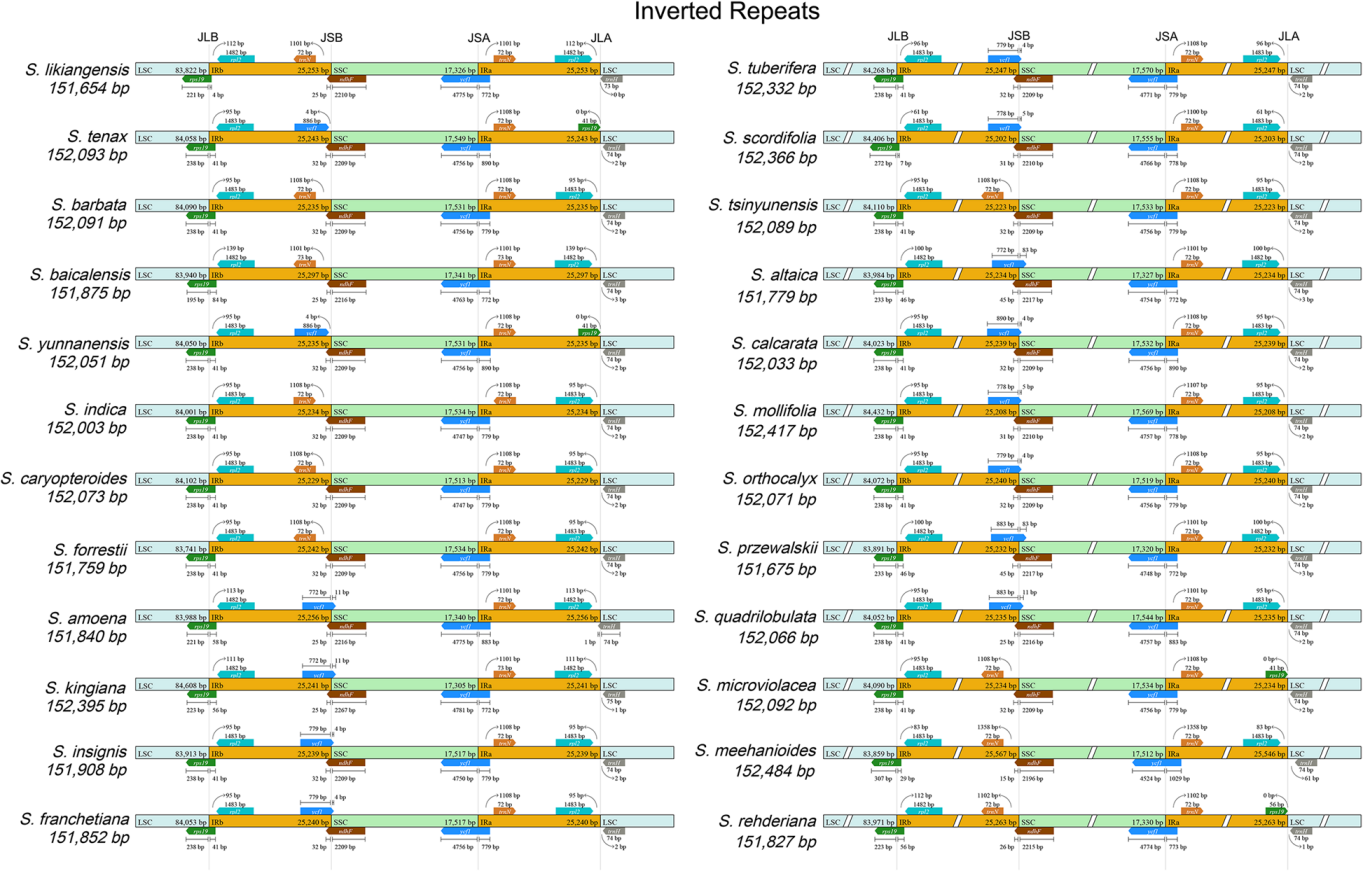
Comparisons of IR, LSC, and SSC regions amongst 24 *Scutellaria* cp genomes. The numbers above denoted the distance between the gene ends, and the region sites, and the numbers below indicate their responding size. The features are not to scale

In the cp genome, the structure is characterized by a circular tetrad composed of the LSC, SSC, and IRs regions. The contraction and expansion of the IRs regions lead to variable genome size [20]. As illustrated in Fig. 3, the structure and connection between IRs regions varied slightly in the 24 *Scutellaria* species. Specifically, a shortened copy of the *rps19* gene was detected at the joint point of the IRb and LSC regions from all the species analyzed. The truncated gene originated in the IRb region and was integrated into the LSC region by 4 - 84 bp fragments. Meanwhile, this pseudogene was also identified in *S. tenax*, *S. yunnanensis*, *S. microviolacea*, and *S. rehderiana*. At the junction of IRb/SSC regions, a truncated copy of *ndhF* gene was inserted into the IRb region with 15 - 45 bp fragments, while *rpl2* was exclusively located in the IRb region. Besides, a truncated copy of *ycf1* gene was observed in all species at the IRa/SSC junction. No *ycf1* pseudogene was found in *S. likiangensis*, *S. barbata*, *S. baicalensis*, *S. indica*, *S. caryopteroides*, *S. forrestii*, *S. tsinyunensis*, *S. microviolacea*, *S. meehanioides*, and *S. rehderiana* in the IRb region. In addition, the *trnN* genes were in the IRa region, while the *trnH* gene of all species was in the LSC. These results indicated that the cp genomes of nine *Scutellaria* species exhibited a distinctive pattern of IRs contraction and expansion.

### Genome comparison and nucleotide diversity

**Fig. 4:**
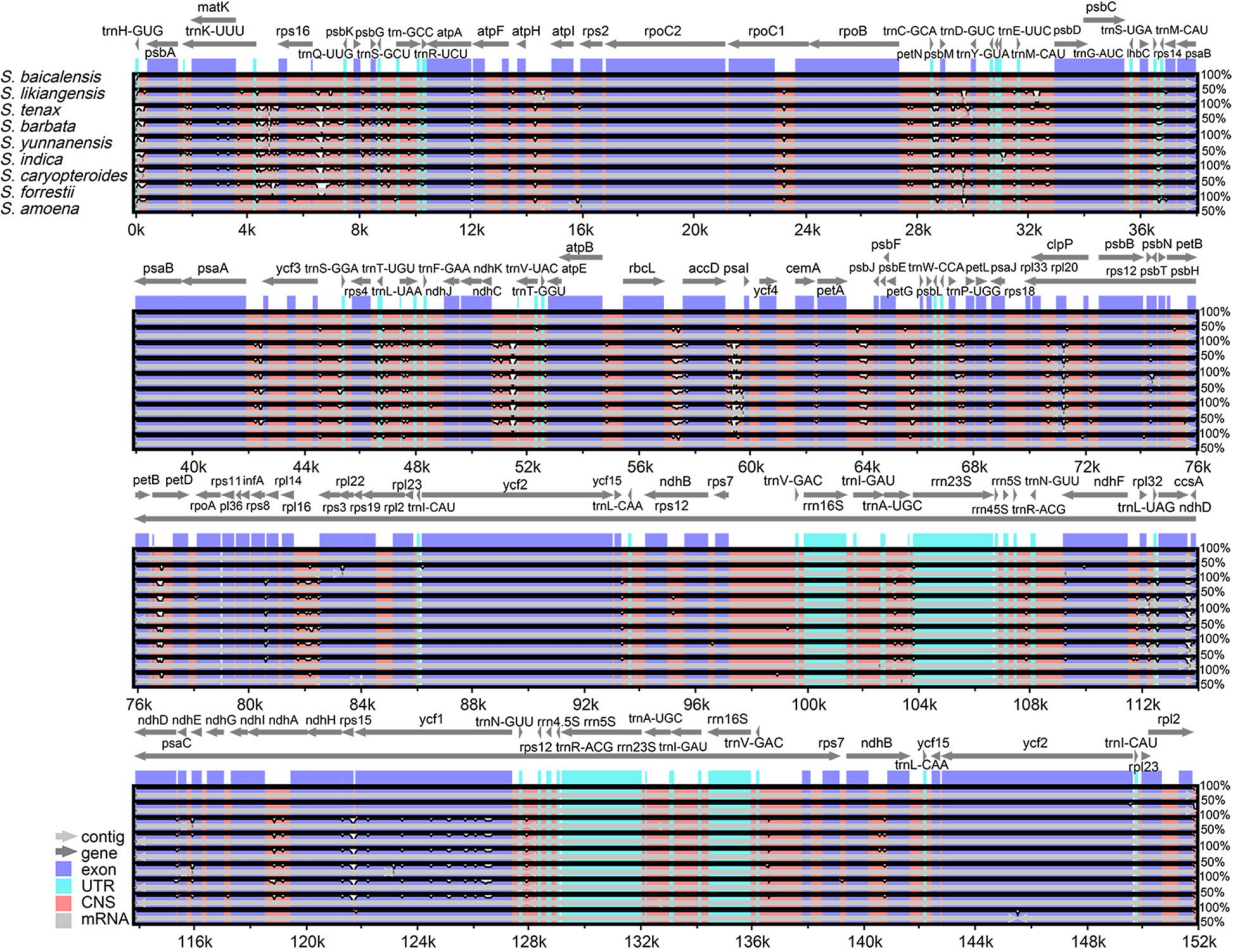
Comparisons of nine cp genomes using *S. baicalensis* (GenBank OP597814) annotation as a reference. The vertical scale ranged from 50% to 100% represents the percentage of identity. Arrows represent the transcriptional direction of each annotated gene

**Fig. 5:**
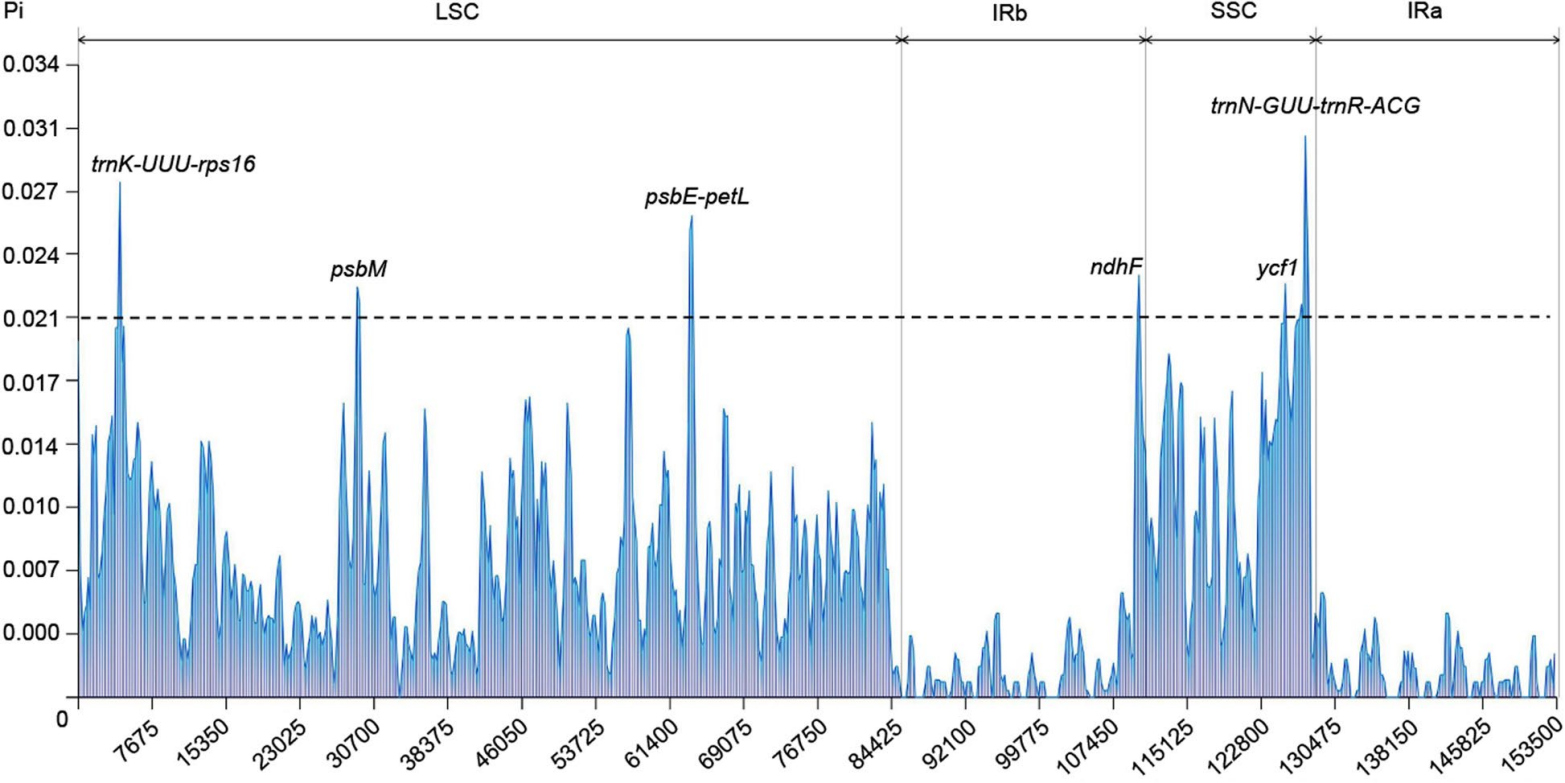
Sliding window analysis of the cp genomes among nine *Scutellaria* species

A comparative analysis was performed to assess the extent of divergence. The findings of this study indicated that the cp genomes of nine *Scutellaria* species were highly conserved, with a greater degree of conservation in the protein-coding regions than in the non-coding regions. Therein, notable mutations were observed in *ycf1* and *petD* genes (Fig. 4). The most differentiation of non-coding regions was observed in the *petN-psbM*, *rps16*-*trnQ-UUG*, *ndhC-trnV-UAC*, *rbcL-accD*, *accD-psaI*, and *rpl16-rps3* intergenic spacers (IGSs). Additionally, the sliding window analysis revealed that the average Pi value of 87 protein-coding genes was 0.0061. Three regions, namely *matK-rps16*, *psbE-petL*, and *trnN-GUU-trnR-ACG*, exhibited highly variable Pi values of > 0.02, with *psbE-petL* displaying the lowest divergence value of 0.026. The *trnN-GUU-trnR-ACG* regions displayed the highest divergence value of 0.031 (Fig. 5). Nine highly polymorphic regions, including *rps16*-*trnQ-UUG*, *petN-psbM*, *ndhC-trnV-UAC*, *rbcL-accD*, *accD-psaI*, *rpl16-rps3*, *matK-rps16*, *psbE-petL*, and *trnN-GUU-trnR-ACG*, were identified as promising molecular markers for distinguish *S. baicalensis* and its contaminants.

### Species authentication analysis

The identification of highly variable regions within the cp genomes represents a valuable resource to distinguish closely related species and provides crucial insights for conducting phylogenetic analyses [22, 23]. An initial ML analysis was conducted on nine IGSs separately, with the default parameters. As shown in Fig. S4, *S. baicalensis* exhibited discernible differentiation from its commonly encountered adulterants based on analysis of *matK-rps16*, *ndhC-trnV-UAC*, *psbE-petL*, and *rps16-trnQ-UUG* loci, whereas the remaining IGSs could not provide sufficient discrimination with low bootstrap values. Therefore, a more comprehensive ML tree was constructed using a combination of these four IGS regions (Fig. S4). In this tree, *S. baicalensis* occupied a distinct branch, indicating the efficacy of these loci in accurately distinguishing it from prevalent contaminants.

Additionally, primers were designed for the nine IGSs (Table S14), and amplification and sequencing experiments were conducted. The results showed that the four IGSs regions (*rps16*-*trnQ-UUG*, *ndhC*-*trnV-UAC*, *matK*-*rps16*, and *psbE*-*petL*) produced products of the expected sizes in selected *Scutellaria* species (Fig. S5). Both amplification and sequencing achieved a 100% success rate, and each species displayed distinct base differences (Fig. S6A-D). In contrast, universal DNA barcodes (ITS, *psbA-trnH*, *matK*, *rbcL*, and *trnL-trnF*) could not identify *S. baicalensis* from its contaminants (Table S15). These findings confirm that the four IGSs are ideal for distinguishing *S. baicalensis* from its adulterants.

### Phylogenetic and divergence time analyses

**Fig. 6:**
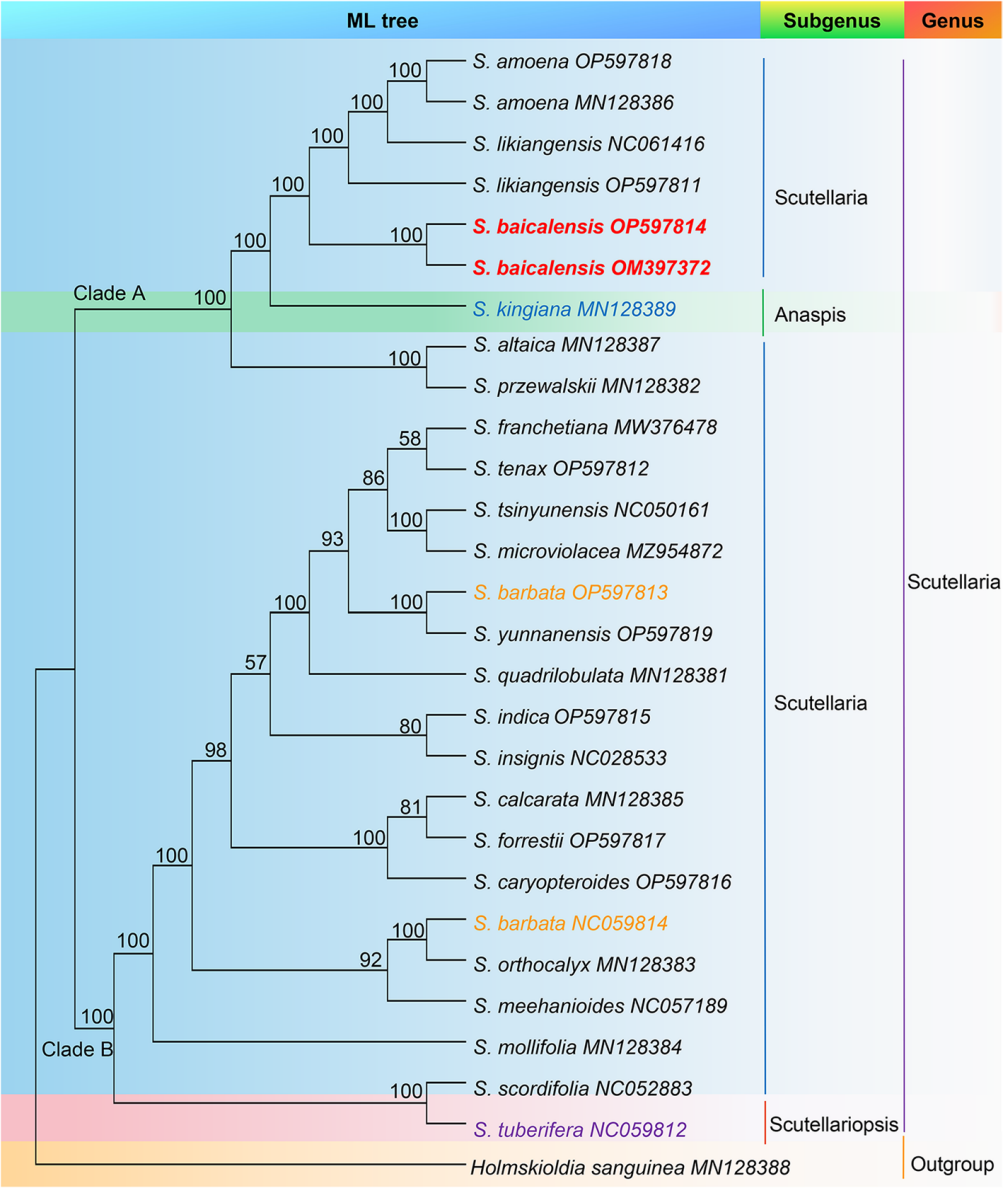
The constructed ML phylogenetic tree among 28 common cp genes from 24 species

Phylogenetic analysis was performed for 28 sequences, including both cp genomes and CDSs, with *Holmskioldia sanguinea* utilized as the outgroup. Most nodes on the phylogenetic tree showed strong support (Fig. 6 and Fig. S7). ML analysis of cp genomes and CDS sequences gave nearly identical topologies with varying levels of support at a few nodes. The involved species were grouped into clades A and B. The former was further divided into the subgenera *Anaspis* and *Scutellaria*, which contained six species, i.e., *S. amoena*, *S. likiangensis*, *S. baicalensis*, *S. altaica*, *S. przewalskii*, and *S. kingiana*. The monophyly of *S. baicalensis* was strongly supported with 100% bootstrap value, suggesting that *S. baicalensis* could be differentiated from its adulterants by the cp genome or shared CDS sequences. Clade B included two subgenera, *Scutellaria* and *Scutellariopsis*, and encompassed the remaining species, indicating relatively weak support for the current division of three subgenera of *Scutellaria*. These results contribute significantly to the taxonomy of *Scutellaria* and may have implications for the identification and conservation of relevant species.

The divergence time estimation showed that clades A and B diverged during the Pliocene Epoch at approximately 4.0 Mya (Fig. S8). Additionally, the most recent common ancestor of the extant subgenera *Scutellaria* and *Anaspis* dates back to the beginning of the late Pleistocene, approximately 2.52 Mya.

## Discussion

### Cp genome organizations

The cp genome is a valuable tool for studying species identification, population genetics, phylogenetics, and gene engineering [20, 24]. The *de novo* assembly and comparative analysis of nine cp genomes of *Scutellaria* were performed in this study, including five species that are being reported for the first time. The size of cp genomes ranged from 151,654 bp to 152,093 bp, mainly resulting from gene insertion and deletion in the non-coding regions. The cp genome sizes of the nine *Scutellaria* species fell within the known range of the genus, ranging from 151,654 bp of *S. likiangensis* to 152,731 bp of *S. baicalensis* [25]. The cp genomes of all species were highly similar in total length, GC content, and gene composition, consistent with earlier findings [7] and similar to other Lamiaceae species. At the genus level, the cp genome of angiosperms was highly conserved [26]. However, the *rpl36*, *psbJ*, *trnfM-CAU*, *trnS-UGA*, *trnG-UCC*, and *trnQ-UUG* genes/IGSs were lost in *S. indica*, suggesting that these variations may be specific to this particular species.

### Codon usage and comparative analyses

Codons are critical in linking genetic materials, amino acids, and proteins within organisms [13]. We found over 90% (RSCU ≧ 1) of *Scutellaria* codons terminated in A/U, and the GC3s value ranged from 38.2% to 39.1%. *Scutellaria* exhibited a high coding efficiency and a strong preference for A/U termination codons, possibly owing to the overall high AT content in the cp genome. This trend was also evident in other angiosperms [13, 15].

Our investigation of *Scutellaria* genomes revealed that noncoding regions displayed more substantial variation than coding regions. Four highly variable regions in non-coding regions identified in this study effectively distinguished the most common *Scutellaria* species. These findings aligned with earlier studies of *Scutellaria* [2]. Non-coding regions evolve rapidly and contain valuable variations for genus phylogenetic analysis [27]. Therefore, the significance of non-coding regions in the cp genome for identifying *Scutellaria* species should be underscored.

### Identification and phylogenetic analysis

Several molecular studies have demonstrated the high identification capability of cp genetic markers in distinguishing *Phyllanthus* and *Isodon* species [15, 28]. Four regions (*matK-rps16*, *ndhC-trnV-UAC*, *psbE-petL*, and *rps16-trnQ-UUG*) have been filtered as candidate molecular markers for discriminating *S. baicalensis* from its common adulterants in this study. The *matK-rps*16 region can distinguish *Triticum* species [29], and *ndhC-trnV-UAC*, *psbE-petL*, and *rps16-trnQ-UUG* have been proposed to identify other species [24, 30]. A possible reason was the different species involved in the screening process, which may have excluded important information. Besides, although universal DNA barcodes, such as *matK*, *psbA-trnH*, and ITS, have been demonstrated to differentiate *S. baicalensis* from closely related species [3, 31, 32], they did not effectively identify common confounding taxa. In contrast, the four identified IGSs were theoretically found to be capable of effectively distinguishing *S. baicalensis* from contaminants, a finding that was further validated in experiments.

The phylogenetic analyses between the cp genome and CDSs showed that *S. baicalensis* occupied an autonomous branch and the sister relationship between *S. baicalensi*s and either *S. kingiana* or *S. amoena* was strongly supported, which agreed with previous findings about ITS and ITS2 regions [8, 32]. Notably, *S. barbata* (Genbank OP597813 and Genbank NC059814) individuals did not cluster and were in different branches, suggesting the intraspecific diversity of *S. barbata*. This finding was reminiscent of a previous study, where the cp genomes of *Isodon rubescens* from diverse geographical regions showed high intraspecific diversity [15]. The variation in the cp sequence of *S. barbata* might also be influenced by the geographical area of origin. Besides, *S. kingiana* and *S. tuberifera*, classified under subgenus *Anaspis* and subgenus *Scutellariopsis* in the Flora of China, respectively, were grouped with subgenus *Scutellaria* in this study. All studied *Scutellaria* species formed a well-supported clade (100%), dividing into two subclades. This challenges the three-subgenus classification proposed in the Flora of China. Notably, the monophyly of subgenus *Anaspis* might be untenable given the limited sample size, consisting of only one species, as aligned with a previous study [7]. Furthermore, most amino acids in CDSs are highly conserved, and phylogenetic analysis based on CDS sequences can be used for phylogenetic studies [33], which agrees with our results. Using cp genome data for classification at the genus or subgenus level has been widely recognized in several plant taxonomic groups [34]. Accordingly, we propose reclassifying the *Scutellaria* into two subgenera, namely, subgenus *Anaspis* and subgenus *Scutellaria*, supported by ITS-based studies [5]. Overall, the monophyly of *S. baicalensis* was verified based on the cp genomes and shared CDS sequences, which provides a basis for studying the species identification, phylogeny, and taxonomy of *Scutellaria*.

### The divergence time of *Scutellaria*

The origins of *Scutellaria* can be traced back to approximately 4.0 Mya based on estimated divergence time, which agreed with previous studies [35, 36]. This timeline coincides with Pliocene, a geological interval from 5.30 to 2.60 Mya, which had a global temperature that was several degrees warmer than current levels. The Pliocene represents a suitable analog for a future anthropogenic greenhouse world [37]. During the early Pliocene, precipitation levels increased significantly, resulting in a warmer and wetter climate that facilitated the expansion of forests in the northern hemisphere and a decrease in global desert areas. These climate changes may influence the migration and diversification of terrestrial plants and lead to an outbreak of species in the ecological niches [38]. Hence, it can be deduced that the climatic conditions prevailing during the Pliocene epoch drove significantly the diversification of *Scutellaria*.

## Conclusion

Here, the cp genomes of nine *Scutellaria* species were *de novo* assembled under the Illumina sequencing platform. Therein, the cp genomes of five species, i.e., *S. indica*, *S. yunnanensis*, *S. tenax*, *S. forrestii*, and *S. caryopteroides*, were reported for the first time. All species had relatively conserved cp genomes with similar genomic structure and gene content. Notably, four identified distinct and highly variable cp loci, namely *matK-rps16*, *ndhC-trnV-UAC*, *psbE-petL*, and *rps16-trnQ-UUG* harbor potential as molecular markers to differentiate *S. baicalensis* from its common dopants. Moreover, the monophyly of *Scutellaria* was confirmed, and both the cp genome-based phylogeny and CDS-based phylogeny proposed its reclassification into two subgenera, *Anaspis* and *Scutellaria*. The divergence time of *Scutellaria* was approximately 4.0 Mya during the Pliocene, and Pliocene climatic conditions are likely a significant factor contributing to the diversification of the genus. Overall, this study provides valuable insights into the safety, effectiveness, and bioprospecting and conservation of *Scutellaria* species.

## Materials and methods

### Plant materials and DNA sequencing

**Fig. 7:**
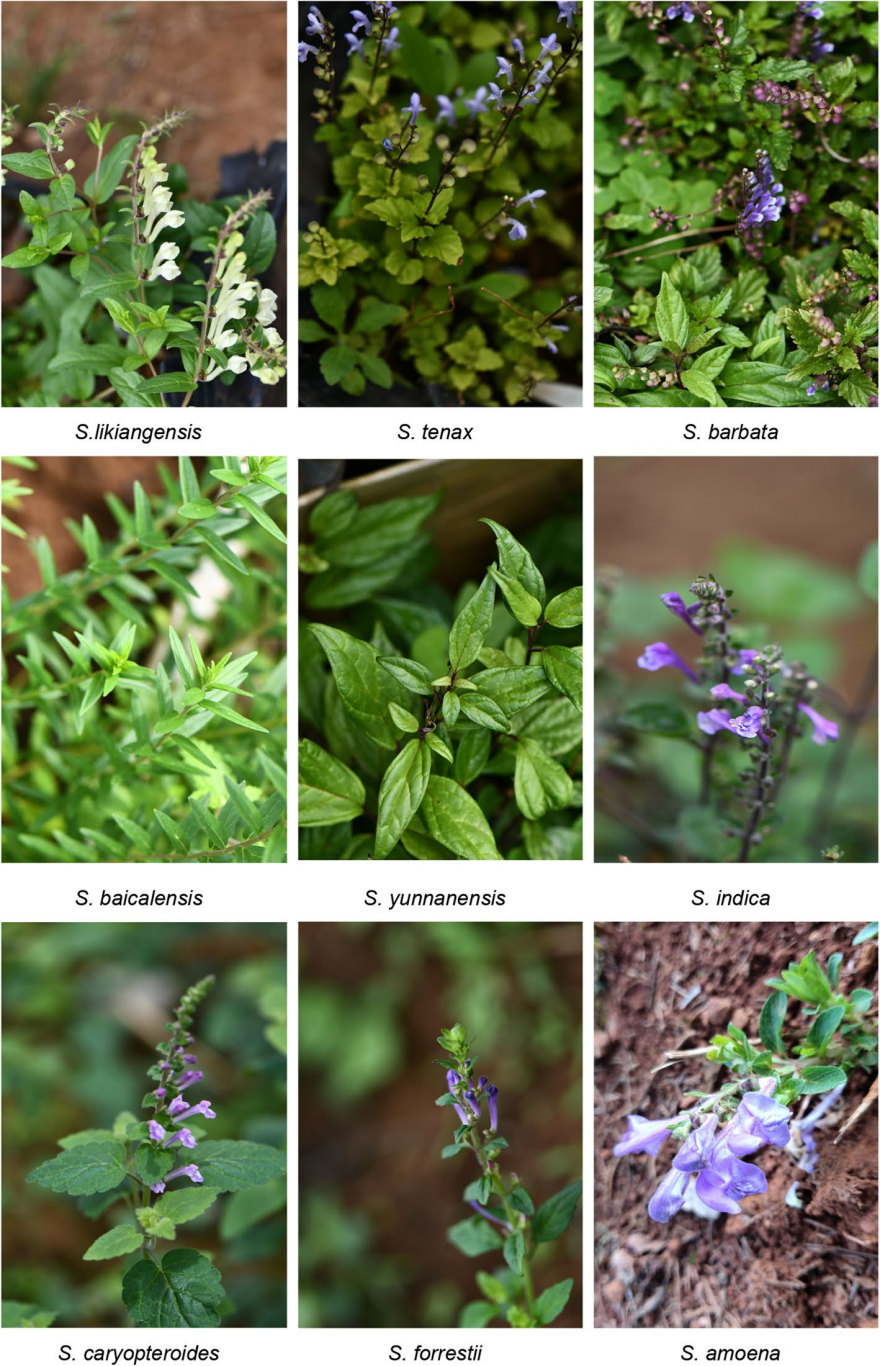
The phenotype of nine *Scutellaria* species

Healthy and fresh leaves from nine *Scutellaria* species, including *S. indica*, *S. yunnanensis*, *S. tenax*, *S. forrestii*, *S. caryopteroides*, *S. baicalensis*, *S. likiangensis*, *S. barbata*, and *S. amoena*, were gathered from the Germplasm Resource Garden, Kunming Zhifen Biotechnology Co., Ltd. in Yunnan, China (24°49′55′′N, 102°48′58″E). The detailed information for each sample was summarized in Fig. 7 and Table S16. All the voucher samples were authenticated by Professor Baozhong Duan and preserved in the herbarium of Dali University. A commercial kit DP305 (Tiangen, Beijing, China) was used to extract the total plant genome DNA. High-quality DNA was used for further sequencing. It is worth noting that PCR amplification studies were restricted to six species (S1-S6, *S. likiangensis, S. tenax, S. barbata, S. baicalensis, S. yunnanensis, S. amoena*) due to the lack of remaining DNA in three *Scutellaria* samples. Additionally, four other species (S10-S13, *S. purpureocardia*, *S. weishanensis*, *S. teniana*, and *S. kingiana*) (Table S16) were included in the PCR amplification experiment.

### Assembly and annotation of *Scutellaria*

Approximately 3 μg DNA was employed to establish the shotgun libraries, sequenced via the Illumina NovaSeq system. NGS QC Toolkit v. 2.3.3 software was utilized to obtain clear reads by trimming paired-end sequence reads. *De novo* assemblies and annotation of the cp genomes were performed by using GetOrganelle v. 1.6.4 and online tools CpGAVAS2 [13, 39] and GeSeq [28, 40] with the parameter sets referred to previous studies [15, 41], respectively. The Apollo rectified intron/exon boundaries and start/stop codons [9, 42]. The cp genomes were mapped through IRscope (https://irscope.shinyapps.io/Chloroplot/). The accession numbers of the annotated cp genome sequences are available on the National Center for Biotechnology Information (NCBI) GenBank database (OP597811-OP597819) (Table 1).

### Repeat sequence and codon preference analyses

The Geneious 9.0.2 software [43] was employed to conduct a GC content analysis. Furthermore, the REPuter program (https://bibiserv.cebitec.uni-bielefeld.de/reputer) was utilized to recognize dispersed repeat sequences, including forward (F), complementary (C), palindromic (P), and reverse (R) [44]. With the setting of >30 bp, ≥90% sequence identity, and a Hamming distance at 3. Simple sequence repeats (SSRs) in the cp genomes were analyzed on the MISA-web (http://pgrc.ipk-gatersleben.de/misa/) [45], and those with different repeat units were regarded as hexanucleotide, pentanucleotide, tetranucleotides, trinucleotides, dinucleotides, mononucleotide, respectively.

Additionally, the amino acid usage frequency and relative synonymous codon usage (RSCU) were identified via the CodonW software [46]. Lastly, TBtools, a software that integrates various biological data handling tools [47], generated a heatmap of the RSCU values.

### Comparative and phylogenetic analyses

The comparative analysis of cp genomes of nine species was conducted using the mVISTA program in Shuffle-LAGAN mode [48], with *S. baicalensis* (GenBank OP597814) as the reference genome. The divergence in the contraction and expansion of the inverted repeat (IR) regions in the cp genomes was visualized by using the IRscope tool (https://irscope.shinyapps.io/irapp/) [49]. The cp genomes were aligned via the MAFFT software (https://mafft.cbrc.jp/alignment/server/index.html) [50]. The nucleotide diversity (Pi) was analyzed using the DnaSP v. 6.12.03 software with previously reported parameter settings [14].

A total of 28 complete cp genome sequences of *Scutellaria,* including 18 reported, were utilized for phylogenetic analysis (Table S17), and five new species were in this study. *Holmskioldia sanguinea* (Genbank MN128388) was selected as an outgroup taxa. Moreover, shared coding sequences (CDSs) were obtained from the complete plastomes, and *S. baicalensis* (GenBank OP597814) serves as the reference chloroplast genome. The alignment of all sequences and trimming were conducted by using MAFFT and TrimAl with the default parameters setting, respectively [50]. The maximum likelihood (ML) tree was constructed using IQ-TREE [51].

### Identification and validation of barcode for species discrimination

The IGS were obtained from nine *Scutellaria* species using PhyloSuite v1.2.2 [52]. Primers were designed based on the variable intergenic regions using Snapgene 6.2.1 (Snapgene, Insightful Science, available at http://www.snapgene.com, last used in 2023). PCR amplifications were conducted in a final volume of 20 μL, comprising 10 μL of 2×Taq Plus PCR Master Mix, 1 μL of each primer, 2 μL of template DNA, and 6 μL of ddH_2_O. All amplifications were performed using a RePure-A PCR system (Applied Biogener, Hangzhou, China) under the following conditions: an initial denaturation at 95 ℃ for 4 min, followed by 40 cycles of 94 ℃ for 30 s, 56 ℃ for 1 min, and 72 ℃ for 1 min, with a final extension at 72 ℃ for 10 min. PCR products were examined by 1% agarose gel electrophoresis to confirm the amplification of the target fragments. The purified PCR products were sequenced in both directions on a 3730XL DNA Sequencer (Applied Biosystems, Waltham, USA) using the same primers at Vazyme Medical Technology (Nanjing, China).

### Divergence time analysis

MEGA X was employed to create a molecular clock tree [53]. The relevant divergence time was then estimated through the TimeTree Resource (http://www.timetree.org/) [28, 54]. Three calibration nodes were employed: (F1) a median time of 2.85-3.85 million years ago (Mya) for the most recent common ancestor (MRCA) of *S. amoena* and *S. baicalensis*; (F2) a median time of 2.85-3.85 Mya for the common ancestor of *S. baicalensis* and *S. barbata*; and (F3) a median time of 0.11-1.11 Mya for the common ancestor of *S. barbata* and *S. indica*.

### Supplementary Information

**Additional file 1:** **Table S1.** Codons in cp genome of *S. likiangensis*. **Table S2.** Codons in cp genome of *S. tenax*. **Table S3.** Codons in cp genome of *S. barbata*. **Table S4.** Codons in cp genome of *S. baicalensis*. **Table S5.** Codons in cp genome of *S. yunnanensis*. **Table S6.** Codons in cp genome of *S. indica*. **Table S7.** Codons in cp genome of *S. caryopteroides*. **Table S8.** Codons in cp genome of *S. forrestii*. **Table S9.** Codons in cp genome of *S. amoena*. **Table S10.** GC content at different positions of CDS sequence codon. **Table S11.** The number of fowarde (F), reverse (R), complementary (C), and palindromic (P) repeats in the cp genome. **Table S12.** The large repeated sequences in the nine Scutel laria cp genomes with diferent hamming distance. F: forward (direct) matching; R: reverse matching;C: complement matching; P: palindromic (inverted) matching. **Table S13.** Number of SSR types in the cp genome. **Table S14.** Primer design by SnapGene. **Table S15.** Universal DNA barcodes primers. **Table S16.** Information about the samples collected. **Table S17.** Species information downloaded by NCBI. **Fig. S1.** The gel electrophoresis results of universal DNA barcodes PCR products. Lane M was the marker of DL2000 Plus. The lanes from left to right corresponded: S1. *S. likiangensis*; S2. *S. barbata*; S3. *S. yunnanensis*; S4. *S. amoena*; S5. *S. tenax*; S6. *S. baicalensis*; S10. *S. purpureocardia*; S11. *S. weishanensis*; S12. *S. teniana*; S13. *S. kingiana*. **Fig. S2.** Phylogenetic tree created using the NJ technique based on the universal DNA barcodes (A. ITS; B. *psbA-trnH*; C.*matK*; D. *rbcL*; E. *trnL-trnF*). **Fig. S3.** A. Repeat sequences detected in *Scutellaria *cp genome. P, F, C, and R indicate the repeat types: R (Reverse repeats), P (Palindromic repeats), F (Forward repeats), C (Complement repeats); B. The number and type of SSRs in *Scutellaria *cp genome. **Fig. S4.** Phylogenetic tree created using the ML technique based on the cp genome's several IGS (A. *accD-psaI*; B.* matK-rps16*; C. *ndhC-trnV-UAC*; D. *petN-psbM*; E. *psbE-petL*; F. *rbcL-accD*; G. *rpl16-rps3*; H. *rps16-trnQ-UUG*; I.* trnN-GUU-trnR-ACG*; J. four IGSs (*matK-rps16*, *ndhC-trnV-UAC*, *psbE-petL*, and *rps16*-*trnQ-UUG*)). **Fig. S5.** The gel electrophoresis results of nine IGS PCR products. Lane M was the marker of DL2000 Plus. The lanes from left to right corresponded: S1. *S. likiangensis*; S2. *S. barbata*; S3. *S. yunnanensis*; S4. *S. amoena*; S5. *S. tenax*; S6. *S. baicalensis*; S10. *S. purpureocardia*; S11. *S. weishanensis*; S12. *S. teniana*; S13. *S. kingiana*. **Fig. S6.** Sequencing chromatograms of the *psbE-petL* barcode in seven *Scutellaria* specvies. **Fig. S7.** The ML phylogenetic tree based on shared coding sequences (CDS) of the 24 species. The bootstrap support values are listed at each node. **Fig. S8.** Divergence times estimation based on cp genomes. The node ages are given for each node.

## Data Availability

The data provided in the study were submitted to the NCBI (https://www.ncbi.nlm.nih.gov/), and accession numbers are shown in Table 1.
