# Supplementary material for: Comparative analysis of medicinal plants Scutellaria baicalensis and common adulterants based on chloroplast genome sequencing
Source: BMC Genomics. 2024 Jan 8;25:39. doi: 10.1186/s12864-023-09920-2 (PMC10773089; doi:10.1186/s12864-023-09920-2)
Supplement: Supplementary file 1 — Additional file 1: Table S1. Codons in cp genome of S. likiangensis. Table S2. Codons in cp genome of S. tenax. Table S3. Codons in cp genome of S. barbata. Table S4. Codons in cp genome of S. baicalensis. Table S5. Codons in cp genome of S. yunnanensis. Table S6. Codons in cp genome of S. indica. Table S7. Codons in cp genome of S. caryopteroides. Table S8. Codons in cp genome of S. forrestii. Table S9. Codons in cp genome of S. amoena. Table S10. GC content at different positions of CDS sequence codon. Table S11. The number of fowarde (F), reverse (R), complementary (C), and palindromic (P) repeats in the cp genome. Table S12. The large repeated sequences in the nine Scutel laria cp genomes with diferent hamming distance. F: forward (direct) matching; R: reverse matching;C: complement matching; P: palindromic (inverted) matching. Table S13. Number of SSR types in the cp genome. Table S14. Primer design by SnapGene. Table S15. Universal DNA barcodes primers. Table S16. Information about the samples collected. Table S17. Species information downloaded by NCBI. Fig. S1. The gel electrophoresis results of universal DNA barcodes PCR products. Lane M was the marker of DL2000 Plus. The lanes from left to right corresponded: S1. S. likiangensis; S2. S. barbata; S3. S. yunnanensis; S4. S. amoena; S5. S. tenax; S6. S. baicalensis; S10. S. purpureocardia; S11. S. weishanensis; S12. S. teniana; S13. S. kingiana. Fig. S2. Phylogenetic tree created using the NJ technique based on the universal DNA barcodes (A. ITS; B. psbA-trnH; C.matK; D. rbcL; E. trnL-trnF). Fig. S3. A. Repeat sequences detected in Scutellaria cp genome. P, F, C, and R indicate the repeat types: R (Reverse repeats), P (Palindromic repeats), F (Forward repeats), C (Complement repeats); B. The number and type of SSRs in Scutellaria cp genome. Fig. S4. Phylogenetic tree created using the ML technique based on the cp genome's several IGS (A. accD-psaI; B. matK-rps16; C. ndhC-trnV-UAC; D. petN-psbM; E. psbE-petL; [file 12864_2023_9920_MOESM1_ESM.pdf]

Table S1. Codons in cp genome of *S. likiangensis*.

Amino acids	Codon	No	RSCU	Amino acids	Codon	No	RSCU	Amino acids	Codon	No	RSCU	Amino acids	Codon	No	RSCU
Ter	UAA	36	1.35		GGC	173	0.4352	Met	AUG	548	1	Ser	AGU	370	1.2803
	UAG	23	0.8625		GGG	296	0.7447	Asn	AAC	235	0.4519		UCA	336	1.1626
	UGA	21	0.7875		GGU	504	1.2679		AAU	805	1.5481		UCC	294	1.0173
Ala	GCA	344	1.0683	His	CAC	133	0.4944	Pro	CCA	280	1.1429		UCG	176	0.609
	GCC	223	0.6925		CAU	405	1.5056		CCC	209	0.8531		UCU	467	1.6159
	GCG	162	0.5031	Ile	AUA	584	0.8985		CCG	139	0.5673	Thr	ACA	347	1.1894
	GCU	559	1.736		AUC	396	0.6092		CCU	352	1.4367		ACC	228	0.7815
Cys	UGC	67	0.5174		AUU	970	1.4923	Gln	CAA	638	1.5448		ACG	122	0.4182
	UGU	192	1.4826	Lys	AAA	899	1.5033		CAG	188	0.4552		ACU	470	1.611
Asp	GAC	167	0.3728		AAG	297	0.4967	Arg	AGA	419	1.806	Val	GUA	470	1.4803
	GAU	729	1.6272	Leu	CUA	351	0.8731		AGG	150	0.6466		GUC	150	0.4724
Glu	GAA	908	1.5416		CUC	146	0.3632		CGA	316	1.3621		GUG	168	0.5291
	GAG	270	0.4584		CUG	168	0.4179		CGC	103	0.444		GUU	482	1.5181
Phe	UUC	425	0.6543		CUU	506	1.2587		CGG	112	0.4828	Trp	UGG	400	1
	UUU	874	1.3457		UUA	748	1.8607		CGU	292	1.2586	Tyr	UAC	157	0.376
Gly	GGA	617	1.5522		UUG	493	1.2264	Ser	AGC	91	0.3149		UAU	678	1.624

Table S2. Codons in cp genome of *S. tenax*.

Amino acids	Codon	No	RSCU	Amino acids	Codon	No	RSCU	Amino acids	Codon	No	RSCU	Amino acids	Codon	No	RSCU
Ter	UAA	48	1.4845		GGC	181	0.4565	Met	AUG	549	1		AGU	374	1.2642
	UAG	22	0.6804		GGG	299	0.7541	Asn	AAC	253	0.4801		UCA	341	1.1527
	UGA	27	0.8351		GGU	494	1.2459		AAU	801	1.5199		UCC	295	0.9972
Ala	GCA	332	1.0498	His	CAC	132	0.4981	Pro	CCA	267	1.1183		UCG	177	0.5983
	GCC	216	0.683		CAU	398	1.5019		CCC	203	0.8503		UCU	489	1.653
	GCG	158	0.4996	Ile	AUA	576	0.8903		CCG	137	0.5738	Thr	ACA	336	1.1497
	GCU	559	1.7676		AUC	396	0.6121		CCU	348	1.4576		ACC	242	0.8281
Cys	UGC	71	0.5299		AUU	969	1.4977	Gln	CAA	631	1.539		ACG	124	0.4243
	UGU	197	1.4701	Lys	AAA	899	1.5071		CAG	189	0.461		ACU	467	1.5979
Asp	GAC	169	0.3802		AAG	294	0.4929	Arg	AGA	424	1.8081	Val	GUA	470	1.498
	GAU	720	1.6198	Leu	CUA	344	0.8647		AGG	154	0.6567		GUC	155	0.494
Glu	GAA	903	1.5331		CUC	149	0.3745		CGA	308	1.3134		GUG	160	0.51
	GAG	275	0.4669		CUG	168	0.4223		CGC	109	0.4648		GUU	470	1.498
Phe	UUC	424	0.6488		CUU	506	1.2719		CGG	115	0.4904	Trp	UGG	399	1
	UUU	883	1.3512		UUA	739	1.8576		CGU	297	1.2665	Tyr	UAC	163	0.3932
Gly	GGA	612	1.5435		UUG	481	1.209	Ser	AGC	99	0.3346		UAU	666	1.6068

Table S3. Codons in cp genome of *S. barbata*.

Amino acids	Codon	No	RSCU	Amino acids	Codon	No	RSCU	Amino acids	Codon	No	RSCU	Amino acids	Codon	No	RSCU
Ter	UAA	47	1.4536		GGC	181	0.4562	Met	AUG	547	1		AGU	373	1.2623
	UAG	23	0.7113		GGG	298	0.7511	Asn	AAC	250	0.4735		UCA	341	1.154
	UGA	27	0.8351		GGU	494	1.2451		AAU	806	1.5265		UCC	297	1.0051
Ala	GCA	332	1.0498	His	CAC	132	0.4981	Pro	CCA	266	1.1118		UCG	177	0.599
	GCC	217	0.6862		CAU	398	1.5019		CCC	201	0.8401		UCU	486	1.6447
	GCG	157	0.4964	Ile	AUA	578	0.8929		CCG	139	0.581	Thr	ACA	333	1.1414
	GCU	559	1.7676		AUC	393	0.6071		CCU	351	1.4671		ACC	243	0.8329
Cys	UGC	71	0.5299		AUU	971	1.5	Gln	CAA	631	1.5371		ACG	127	0.4353
	UGU	197	1.4701	Lys	AAA	902	1.5134		CAG	190	0.4629		ACU	464	1.5904
Asp	GAC	169	0.3806		AAG	290	0.4866	Arg	AGA	424	1.8107	Val	GUA	470	1.5016
	GAU	719	1.6194	Leu	CUA	345	0.8676		AGG	153	0.6534		GUC	152	0.4856
Glu	GAA	901	1.531		CUC	150	0.3772		CGA	306	1.3068		GUG	161	0.5144
	GAG	276	0.469		CUG	169	0.425		CGC	110	0.4698		GUU	469	1.4984
Phe	UUC	422	0.6448		CUU	504	1.2674		CGG	115	0.4911	Trp	UGG	399	1
	UUU	887	1.3552		UUA	740	1.8609		CGU	297	1.2683	Tyr	UAC	162	0.3913
Gly	GGA	614	1.5476		UUG	478	1.202	Ser	AGC	99	0.335		UAU	666	1.6087

Table S4. Codons in cp genome of *S. baicalensis*.

Amino acids	Codon	No	RSCU	Amino acids	Codon	No	RSCU	Amino acids	Codon	No	RSCU	Amino acids	Codon	No	RSCU
Ter	UAA	38	1.425		GGC	170	0.4288	Met	AUG	545	1		AGU	370	1.2832
	UAG	23	0.8625		GGG	296	0.7465	Asn	AAC	238	0.4551		UCA	336	1.1653
	UGA	19	0.7125		GGU	504	1.2711		AAU	808	1.5449		UCC	295	1.0231
Ala	GCA	344	1.0667	His	CAC	131	0.4897	Pro	CCA	282	1.1463		UCG	170	0.5896
	GCC	222	0.6884		CAU	404	1.5103		CCC	207	0.8415		UCU	466	1.6162
	GCG	163	0.5054	Ile	AUA	581	0.8943		CCG	142	0.5772	Thr	ACA	344	1.1831
	GCU	561	1.7395		AUC	401	0.6172		CCU	353	1.435		ACC	226	0.7773
Cys	UGC	68	0.5271		AUU	967	1.4885	Gln	CAA	641	1.5427		ACG	120	0.4127
	UGU	190	1.4729	Lys	AAA	899	1.5109		CAG	190	0.4573		ACU	473	1.6268
Asp	GAC	168	0.3746		AAG	291	0.4891	Arg	AGA	423	1.8207	Val	GUA	474	1.4929
	GAU	729	1.6254	Leu	CUA	349	0.8696		AGG	150	0.6456		GUC	148	0.4661
Glu	GAA	899	1.5368		CUC	146	0.3638		CGA	315	1.3558		GUG	166	0.5228
	GAG	271	0.4632		CUG	167	0.4161		CGC	102	0.439		GUU	482	1.5181
Phe	UUC	426	0.6569		CUU	506	1.2608		CGG	112	0.4821	Trp	UGG	398	1
	UUU	871	1.3431		UUA	748	1.8638		CGU	292	1.2568	Tyr	UAC	157	0.3774
Gly	GGA	616	1.5536		UUG	492	1.2259	Ser	AGC	93	0.3225		UAU	675	1.6226

Table S5. Codons in cp genome of *S. yunnanensis*.

Amino acids	Codon	No	RSCU	Amino acids	Codon	No	RSCU	Amino acids	Codon	No	RSCU	Amino acids	Codon	No	RSCU
Ter	UAA	47	1.4536		GGC	181	0.4562	Met	AUG	547	1		AGU	374	1.2649
	UAG	23	0.7113		GGG	298	0.7511	Asn	AAC	250	0.4739		UCA	341	1.1533
	UGA	27	0.8351		GGU	494	1.2451		AAU	805	1.5261		UCC	297	1.0045
Ala	GCA	332	1.0498	His	CAC	132	0.4981	Pro	CCA	266	1.1118		UCG	177	0.5986
	GCC	217	0.6862		CAU	398	1.5019		CCC	202	0.8443		UCU	486	1.6437
	GCG	157	0.4964	Ile	AUA	577	0.8918		CCG	139	0.581	Thr	ACA	334	1.1438
	GCU	559	1.7676		AUC	393	0.6074		CCU	350	1.4629		ACC	244	0.8356
Cys	UGC	71	0.5299		AUU	971	1.5008	Gln	CAA	631	1.5371		ACG	127	0.4349
	UGU	197	1.4701	Lys	AAA	901	1.513		CAG	190	0.4629		ACU	463	1.5856
Asp	GAC	169	0.3806		AAG	290	0.487	Arg	AGA	424	1.8107	Val	GUA	471	1.5036
	GAU	719	1.6194	Leu	CUA	345	0.8676		AGG	153	0.6534		GUC	152	0.4852
Glu	GAA	901	1.531		CUC	150	0.3772		CGA	307	1.311		GUG	161	0.514
	GAG	276	0.469		CUG	169	0.425		CGC	109	0.4655		GUU	469	1.4972
Phe	UUC	422	0.6443		CUU	504	1.2674		CGG	115	0.4911	Trp	UGG	399	1
	UUU	888	1.3557		UUA	740	1.8609		CGU	297	1.2683	Tyr	UAC	162	0.3918
Gly	GGA	614	1.5476		UUG	478	1.202	Ser	AGC	99	0.3348		UAU	665	1.6082

Table S6. Codons in cp genome of *S. indica*.

Amino acids	Codon	No	RSCU	Amino acids	Codon	No	RSCU	Amino acids	Codon	No	RSCU	Amino acids	Codon	No	RSCU
Ter	UAA	40	1.5		GGC	180	0.4523	Met	AUG	552	1		AGU	366	1.2585
	UAG	21	0.7875		GGG	294	0.7387	Asn	AAC	245	0.4653		UCA	342	1.1759
	UGA	19	0.7125		GGU	503	1.2638		AAU	808	1.5347		UCC	290	0.9971
Ala	GCA	339	1.0585	His	CAC	130	0.4887	Pro	CCA	274	1.1276		UCG	174	0.5983
	GCC	218	0.6807		CAU	402	1.5113		CCC	211	0.8683		UCU	480	1.6504
	GCG	164	0.5121	Ile	AUA	577	0.8904		CCG	138	0.5679	Thr	ACA	340	1.1644
	GCU	560	1.7486		AUC	394	0.608		CCU	349	1.4362		ACC	236	0.8082
Cys	UGC	66	0.5116		AUU	973	1.5015	Gln	CAA	635	1.5375		ACG	124	0.4247
	UGU	192	1.4884	Lys	AAA	908	1.5058		CAG	191	0.4625		ACU	468	1.6027
Asp	GAC	169	0.3764		AAG	298	0.4942	Arg	AGA	423	1.8194	Val	GUA	479	1.5051
	GAU	729	1.6236	Leu	CUA	351	0.8699		AGG	149	0.6409		GUC	156	0.4902
Glu	GAA	907	1.5308		CUC	151	0.3742		CGA	309	1.329		GUG	161	0.5059
	GAG	278	0.4692		CUG	169	0.4188		CGC	107	0.4602		GUU	477	1.4988
Phe	UUC	426	0.6509		CUU	512	1.2689		CGG	113	0.486	Trp	UGG	399	1
	UUU	883	1.3491		UUA	749	1.8563		CGU	294	1.2645	Tyr	UAC	160	0.386
Gly	GGA	615	1.5452		UUG	489	1.2119	Ser	AGC	93	0.3198		UAU	669	1.614

Table S7. Codons in cp genome of *S. caryopteroides*.

Amino acids	Codon	No	RSCU	Amino acids	Codon	No	RSCU	Amino acids	Codon	No	RSCU	Amino acids	Codon	No	RSCU
Ter	UAA	41	1.5375		GGC	178	0.4475	Met	AUG	551	1		AGU	367	1.2626
	UAG	21	0.7875		GGG	292	0.7341	Asn	AAC	244	0.463		UCA	337	1.1594
	UGA	18	0.675		GGU	505	1.2696		AAU	810	1.537		UCC	291	1.0011
Ala	GCA	339	1.0594	His	CAC	132	0.4944	Pro	CCA	276	1.1358		UCG	174	0.5986
	GCC	216	0.675		CAU	402	1.5056		CCC	207	0.8519		UCU	481	1.6548
	GCG	165	0.5156	Ile	AUA	577	0.8909		CCG	139	0.572	Thr	ACA	344	1.1761
	GCU	560	1.75		AUC	395	0.6099		CCU	350	1.4403		ACC	235	0.8034
Cys	UGC	66	0.5116		AUU	971	1.4992	Gln	CAA	634	1.5426		ACG	124	0.4239
	UGU	192	1.4884	Lys	AAA	906	1.505		CAG	188	0.4574		ACU	467	1.5966
Asp	GAC	169	0.3768		AAG	298	0.495	Arg	AGA	424	1.8223	Val	GUA	480	1.5035
	GAU	728	1.6232	Leu	CUA	348	0.8635		AGG	148	0.6361		GUC	155	0.4855
Glu	GAA	909	1.5316		CUC	152	0.3772		CGA	309	1.3281		GUG	163	0.5106
	GAG	278	0.4684		CUG	170	0.4218		CGC	107	0.4599		GUU	479	1.5004
Phe	UUC	429	0.654		CUU	510	1.2655		CGG	114	0.49	Trp	UGG	399	1
	UUU	883	1.346		UUA	749	1.8586		CGU	294	1.2636	Tyr	UAC	159	0.3813
Gly	GGA	616	1.5487		UUG	489	1.2134	Ser	AGC	94	0.3234		UAU	675	1.6187

Table S8. Codons in cp genome of *S. forrestii*.

Amino acids	Codon	No	RSCU	Amino acids	Codon	No	RSCU	Amino acids	Codon	No	RSCU	Amino acids	Codon	No	RSCU
Ter	UAA	40	1.5		GGC	181	0.4545	Met	AUG	551	1		AGU	367	1.2619
	UAG	21	0.7875		GGG	293	0.7357	Asn	AAC	243	0.4624		UCA	338	1.1622
	UGA	19	0.7125		GGU	504	1.2655		AAU	808	1.5376		UCC	291	1.0006
Ala	GCA	342	1.0679	His	CAC	131	0.4925	Pro	CCA	275	1.134		UCG	175	0.6017
	GCC	216	0.6745		CAU	401	1.5075		CCC	206	0.8495		UCU	480	1.6504
	GCG	163	0.509	Ile	AUA	578	0.8915		CCG	139	0.5732	Thr	ACA	345	1.1795
	GCU	560	1.7486		AUC	395	0.6093		CCU	350	1.4433		ACC	235	0.8034
Cys	UGC	66	0.5116		AUU	972	1.4992	Gln	CAA	635	1.5375		ACG	124	0.4239
	UGU	192	1.4884	Lys	AAA	909	1.505		CAG	191	0.4625		ACU	466	1.5932
Asp	GAC	169	0.3764		AAG	299	0.495	Arg	AGA	423	1.8194	Val	GUA	481	1.5102
	GAU	729	1.6236	Leu	CUA	344	0.8554		AGG	148	0.6366		GUC	152	0.4772
Glu	GAA	909	1.5316		CUC	151	0.3755		CGA	309	1.329		GUG	162	0.5086
	GAG	278	0.4684		CUG	170	0.4227		CGC	107	0.4602		GUU	479	1.5039
Phe	UUC	428	0.6514		CUU	512	1.2731		CGG	114	0.4903	Trp	UGG	399	1
	UUU	886	1.3486		UUA	748	1.8599		CGU	294	1.2645	Tyr	UAC	160	0.3851
Gly	GGA	615	1.5443		UUG	488	1.2134	Ser	AGC	94	0.3232		UAU	671	1.6149

Table S9. Codons in cp genome of *S. amoena*.

Amino acids	Codon	No	RSCU	Amino acids	Codon	No	RSCU	Amino acids	Codon	No	RSCU	Amino acids	Codon	No	RSCU
Ter	UAA	38	1.425		GGC	172	0.4319	Met	AUG	550	1		AGU	371	1.2845
	UAG	21	0.7875		GGG	298	0.7483	Asn	AAC	236	0.4525		UCA	333	1.1529
	UGA	21	0.7875		GGU	504	1.2655		AAU	807	1.5475		UCC	294	1.0179
Ala	GCA	348	1.0799	His	CAC	133	0.4963	Pro	CCA	284	1.1556		UCG	176	0.6093
	GCC	223	0.692		CAU	403	1.5037		CCC	209	0.8505		UCU	465	1.6099
	GCG	161	0.4996	Ile	AUA	580	0.8905		CCG	140	0.5697	Thr	ACA	346	1.191
	GCU	557	1.7285		AUC	398	0.6111		CCU	350	1.4242		ACC	228	0.7849
Cys	UGC	68	0.5251		AUU	976	1.4985	Gln	CAA	637	1.5386		ACG	120	0.4131
	UGU	191	1.4749	Lys	AAA	903	1.5063		CAG	191	0.4614		ACU	468	1.611
Asp	GAC	167	0.3703		AAG	296	0.4937	Arg	AGA	418	1.8017	Val	GUA	474	1.4941
	GAU	735	1.6297	Leu	CUA	352	0.8731		AGG	150	0.6466		GUC	149	0.4697
Glu	GAA	908	1.5442		CUC	149	0.3696		CGA	320	1.3793		GUG	165	0.5201
	GAG	268	0.4558		CUG	166	0.4117		CGC	104	0.4483		GUU	481	1.5162
Phe	UUC	425	0.6569		CUU	505	1.2526		CGG	110	0.4741	Trp	UGG	400	1
	UUU	869	1.3431		UUA	751	1.8628		CGU	290	1.25	Tyr	UAC	157	0.3747
Gly	GGA	619	1.5543		UUG	496	1.2303	Ser	AGC	94	0.3254		UAU	681	1.6253

Table S10. GC content at different positions of CDS sequence codon.

Species	codon No.	GC1	GC2	GC3	GC_all	ENC	GC3s
S. likiangensis	50551	38.11	38.65	38.33	38.36	55.48	38.3
S. tenax	50697	38.40	37.75	38.95	38.37	55.90	38.9
S. barbata	50697	38.04	37.87	39.11	38.34	55.81	39.1
S. baicalensis	50625	38.68	38.04	38.27	38.33	55.73	38.3
S. yunnanensis	50683	38.26	38.48	38.30	38.35	55.63	38.3
S. indica	50667	37.57	38.93	38.56	38.35	55.59	38.4
S. caryopteroides	50691	38.94	37.69	38.44	38.36	55.61	38.4
S. forrestii	50586	38.55	38.23	38.34	38.38	55.54	38.2
S. amoena	50613	37.93	38.35	38.77	38.35	55.67	38.8
all	455813	38.46	38.47	38.13	38.35	55.47	38.0

Table S11. The number of fowarde (F), reverse (R), complementary (C), and palindromic (P) repeats in the cp genome.

Species	F						P						R						C					
	30-39	40-49	50-59	60-69	≥70	Total	30-39	40-49	50-59	60-69	≥70	Total	30-39	40-49	50-59	60-69	≥70	Total	30-39	40-49	50-59	60-69	≥70	Total
S. likiangensis	12	1	1	1	4	19	10	2	0	1	2	15	0	0	0	0	0	0	0	0	0	0	0	0
S. tenax	10	1	0	0	0	11	11	3	1	2	1	18	0	0	0	0	0	0	0	0	0	0	0	0
S. barbata	12	1	1	0	0	14	10	2	1	1	1	15	0	0	0	0	0	0	0	0	0	0	0	0
S. baicalensis	12	2	0	0	0	14	11	2	0	1	2	16	1	0	0	0	0	1	0	0	0	0	0	0
S. yunnanensis	11	1	0	0	0	12	10	2	1	1	1	15	0	0	0	0	0	0	0	0	0	0	0	0
S. indica	11	2	0	0	0	13	10	3	0	1	1	15	0	0	0	0	0	0	0	0	0	0	0	0
S. caryopteroides	12	1	1	1	0	15	10	3	1	1	1	16	0	0	0	0	0	0	0	0	0	0	0	0
S. forrestii	11	1	1	1	0	14	11	3	1	1	1	17	0	0	0	0	0	0	0	0	0	0	0	0
S. amoena	12	1	1	1	1	16	13	2	0	1	1	17	0	0	0	0	0	0	0	0	0	0	0	0

Table S12. The large repeated sequences in the nine *Scutellaria* cp genomes with diferent hamming distance. **F**: forward (direct) matching; **R**: reverse matching;C: complement matching; **P**: palindromic (inverted) matching.

Species	Hamming Distance=3					Hamming Distance=2					Hamming Distance=1				
	**F**	**P**	**R**	**C**	**all**	**F**	**P**	**R**	**C**	**all**	**F**	**P**	**R**	**C**	**all**
S. likiangensis	19	15	0	0	34	9	8	0	0	17	8	7	0	0	15
S. tenax	11	18	0	0	29	1	9	0	0	10	6	1	0	0	7
S. barbata	14	15	0	0	29	5	8	0	0	13	3	6	0	0	9
S. baicalensis	14	16	1	0	31	3	9	1	0	13	2	7	0	0	9
S. yunnanensis	12	15	0	0	27	2	8	0	0	10	1	6	0	0	7
S. indica	13	15	0	0	28	4	7	0	0	11	3	5	0	0	8
S. caryopteroides	15	16	0	0	31	6	8	0	0	14	5	6	0	0	11
S. forrestii	14	17	0	0	31	5	8	0	0	13	4	6	0	0	10
S. amoena	16	17	0	0	33	6	9	0	0	15	5	5	0	0	10
Total repeated sequences	128	144	1	0	273	41	74	0	0	115	37	49	0	0	86

Table S13. Number of SSR types in the cp genome.

Species	mono-	di-	tri-	tetra-	penta-	hexa-	Total
S. likiangensis	18	6	3	4	2	0	33
S. tenax	26	6	4	7	0	1	44
S. barbata	22	5	3	4	0	0	34
S. baicalensis	17	4	1	4	0	5	31
S. yunnanensis	27	6	4	7	0	0	44
S. indica	23	6	3	4	0	0	36
S. caryopteroides	18	5	3	4	1	2	33
S. forrestii	24	6	3	4	0	0	37
S. amoena	26	5	4	6	0	0	41

Table S14. Primer design by SnapGene

Fragment	Primer ID	Base sequence (5'-3′)	Length/bp	Tm/ºC
petN-psbM	pp F	AAGTACTACTAATTGAGTTCAGGA	24	53
	pp R	CAGAAGGAAGGTTTCATTCAATTG	24	55
ndhC-trnV-UAC	ntu F	AGACGCACTCCTATGAAC	18	53
	ntu R	CAGTCAGTTACAAGCAACAAAC	22	55
rbcL-accD	ra F	GAAAAGTAATTACTCTCCGTTCTC	24	54
	ra R	ATTTTTTATACAAAGAGGGGGCAC	24	56
accD-psaI	ap F	AGCGCACTAAGTTCAATTTTTTTA	24	54
	ap R	CAAATAGTAAATCGAGGTACCCC	23	56
rpl16-rps3	rr F	GTTATAGTTGATGGTTGTTTCGG	23	54
	rr R	AAGGAAAAAACAATGAACGTTTTC	24	54
matK-rps16	mr F	CCTCGGAGACAGGTAAATTTATGA	24	56
	mr R	TCACTACCCCGGGATTTCT	19	56
psbE-petL	ppl F	GCTCAGCTCCACATATTCTTG	21	56
	ppl R	AAATACCATATTTCATTTAGCCCC	24	54
trnN-GUU-trnR-ACG	tta F	CATTCTGAATTAACCAATTCAGA	23	52
	tta R	GTACTTCCCTTTTGGGCC	18	54
rps16-trnQ-UUG	rtu F	AACATTCCTATAATTTGGAACCGG	24	56
	rtu R	TGGAGCATAAAAAATGGATCTACG	24	56

Table S15. Universal DNA barcodes primers

Fragment	Primer ID	Base sequence (5'-3′)	Length/bp	Tm/°C
ITS	2F	ATGCGATACTTGGTGTGAAT	20	56
	3R	GACGCTTCTCCAGACTACAAT	21	56
psbA-trnH	PA	GTTATGCATGAACGTAATGCTC	22	55
	TH	CGCGCATGGTGGATTCACAATCC	23	55
matK	matK F	CCTATCCATCTGGAAATCTTAG	22	55
	matK R	GTTCTAGCACAAGAAAGTCG	20	55
rbcL	rbcL F	ATGTCACCACAAACAGAAACT	21	55
	rbcL R	CGGTACCAGCGTGAATATGAT	21	55
trnL-trnF	trnL F	CGAAATCGGTAGACGCTACG	20	54
	trnF R	ATTTGAACTGGTGACACGAG	20	54

Table S16. Information about the samples collected

No.	Species	Locality	Voucher specimen
S1	*S. likiangensis*	Kunming, Yunnan, China	HQ202201
S2	*S. barbata*	Kunming, Yunnan, China	HQ202202
S3	*S. yunnanensis*	Kunming, Yunnan, China	HQ202203
S4	*S. amoena*	Kunming, Yunnan, China	HQ202204
S5	*S. tenax*	Kunming, Yunnan, China	HQ202205
S6	*S. baicalensis*	Kunming, Yunnan, China	HQ202206
S7	*S. indica*	Kunming, Yunnan, China	HQ202207
S8	*S. caryopteroides*	Kunming, Yunnan, China	HQ202208
S9	*S. forrestii*	Kunming, Yunnan, China	HQ202209
S10	*S. purpureocardia*	Kunming, Yunnan, China	HQ202210
S11	*S. weishanensis*	Kunming, Yunnan, China	HQ202211
S12	*S. teniana*	Kunming, Yunnan, China	HQ202210
S13	*S. kingiana*	Kunming, Yunnan, China	HQ202213

Table S17. Species information downloaded by NCBI.

No.	Species	GenBank accession	No.	Species	GenBank accession
1	*S. insignis*	NC028533	12	*S. mollifolia*	MN128384
2	*S. kingiana*	MN128389	13	*S. orthocalyx*	MN128383
3	*S. baicalensis*	OM397372	14	*S. przewalskii*	MN128382
4	*S. barbata*	NC059814	15	*S. quadrilobulata*	MN128381
5	*S. tuberifera*	NC059812	16	*S. microviolacea*	MZ954872
6	*S. franchetiana*	MW376478	17	*S. meehanioides*	NC057189
7	*S. scordifolia*	NC052883	18	*S. likiangensis*	NC061416
8	*S. tsinyunensis*	NC050161	19	*Holmskioldia sanguinea*	MN128388
9	*S. altaica*	MN128387			
10	*S. amoena*	MN128386			
11	*S. calcarata*	MN128385			

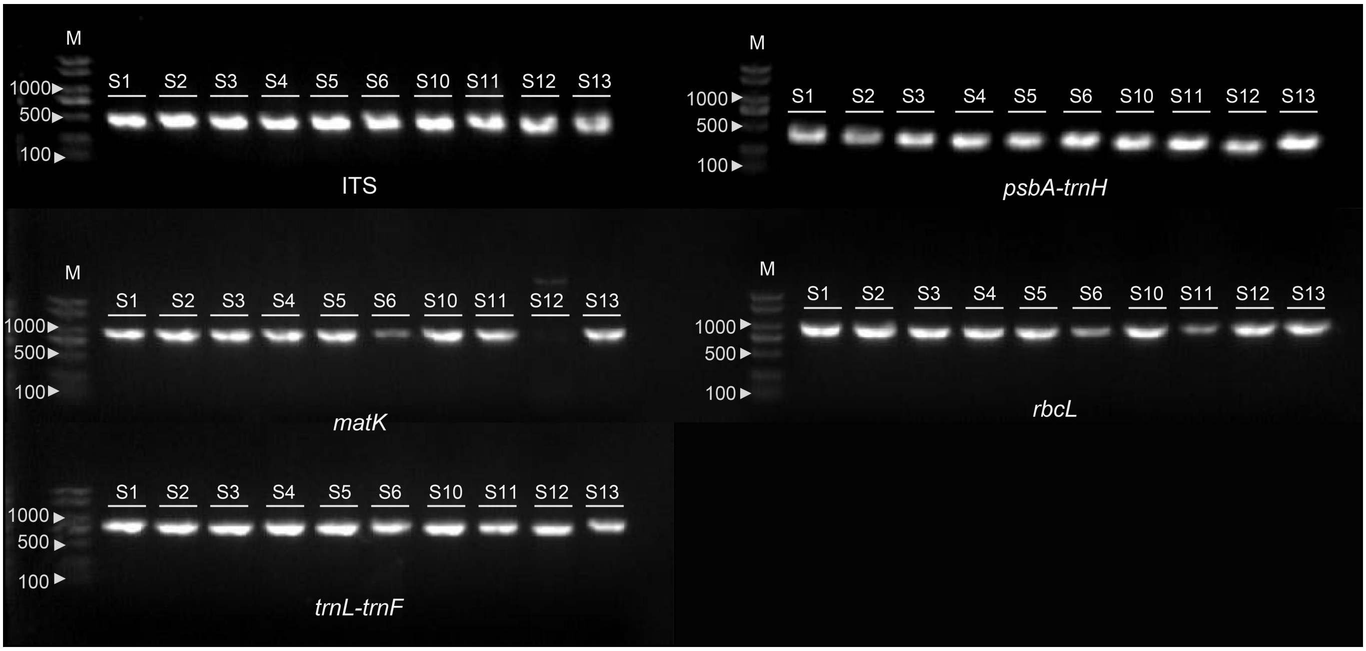

Fig. S1.The gel electrophoresis results of universal DNA barcodes PCR products. Lane M was the marker of DL2000 Plus. The lanes from left to right corresponded: S1. *S. likiangensis*; S2. *S. barbata*; S3. *S. yunnanensis*; S4. *S. amoena*; S5. *S. tenax*; S6. *S. baicalensis*; S10. *S. purpureocardia*; S11. *S. weishanensis*; S12. *S. teniana*; S13. *S. kingiana*.

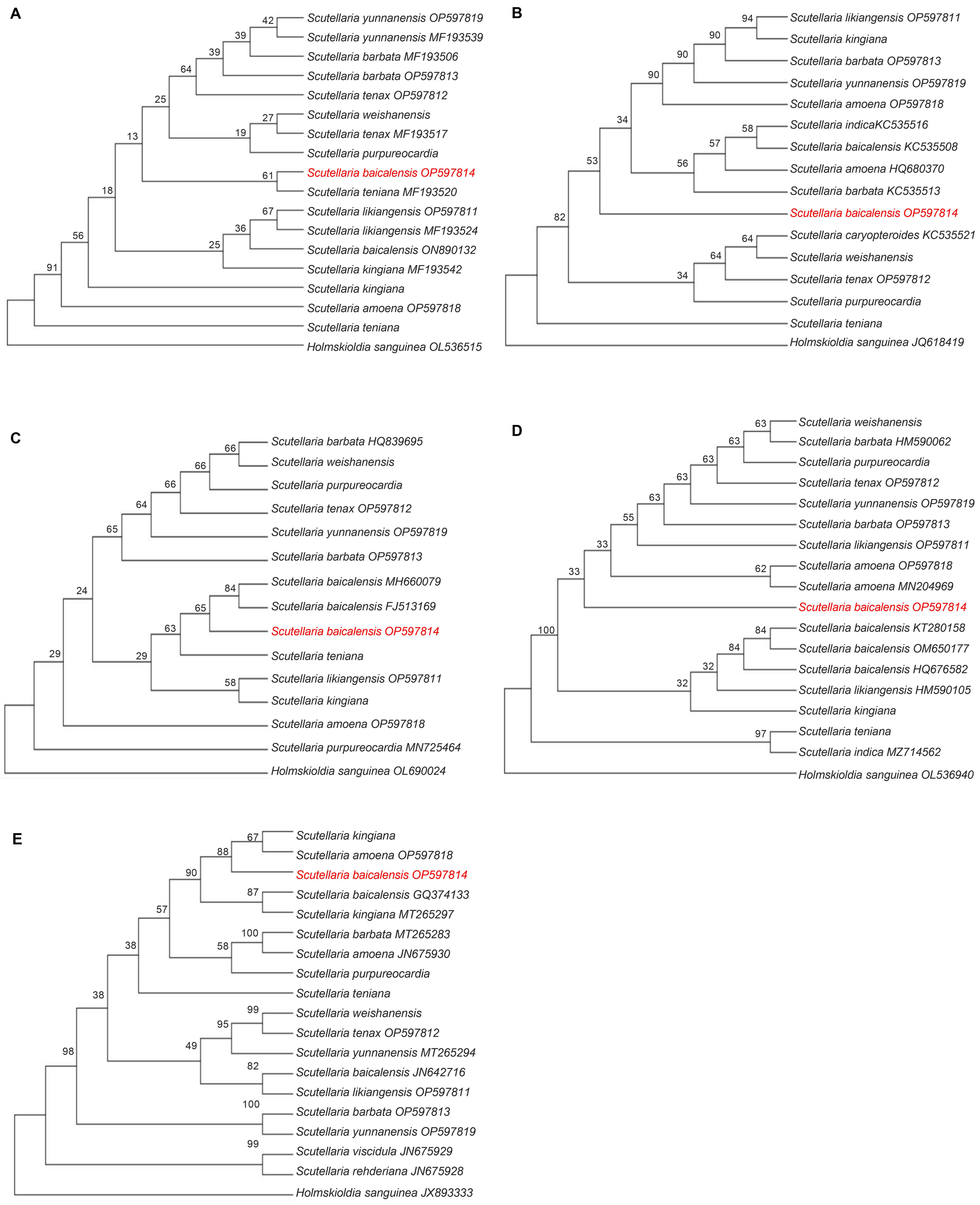

Fig. S2. Phylogenetic tree created using the NJ technique based on the universal DNA barcodes (**A**. ITS; **B**. *psbA-trnH*; **C**.*matK*; **D**. *rbcL*; **E.** *trnL-trnF*)

A

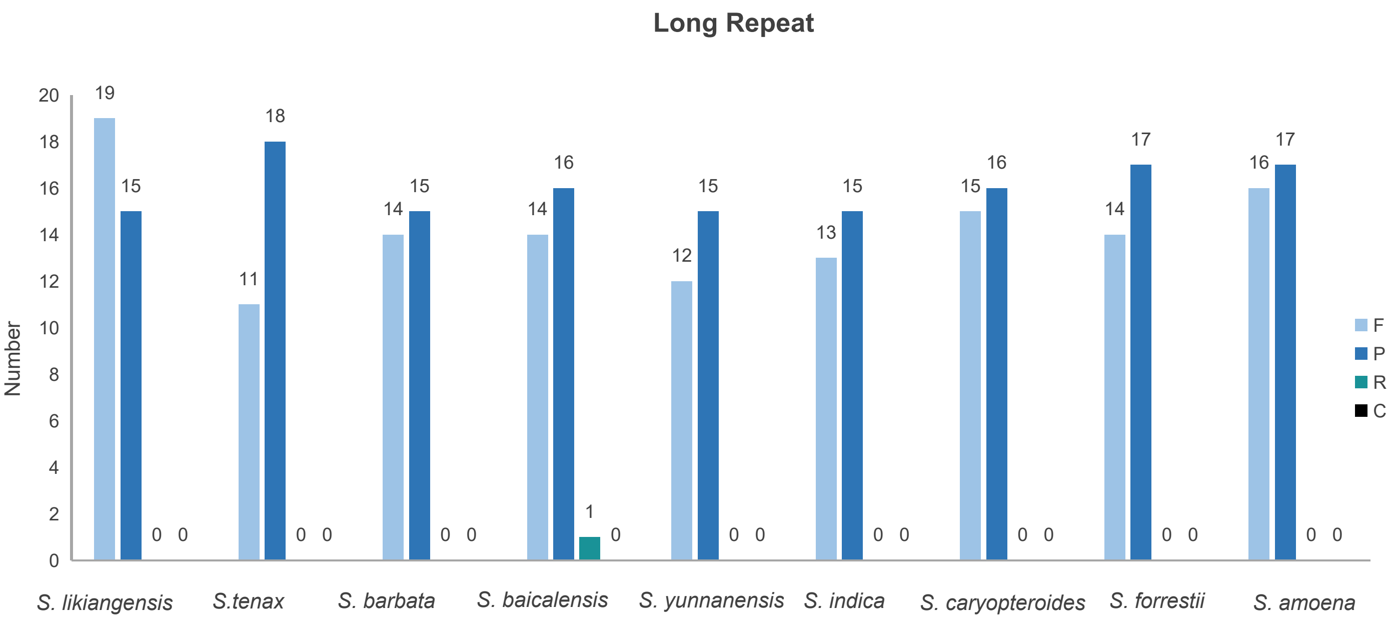

B

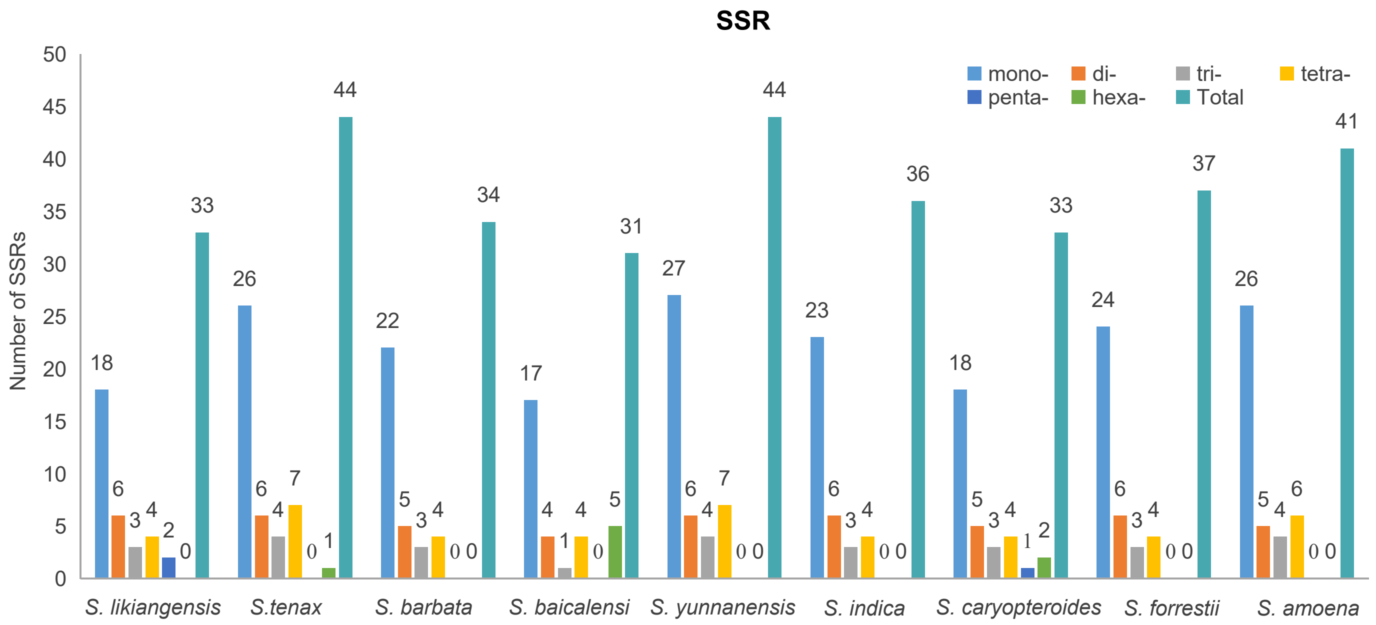

Fig. S3 A. Repeat sequences detected in *Scutellaria* cp genome. P, F, C, and R indicate the repeat types: R (Reverse repeats), P (Palindromic repeats), F (Forward repeats), C (Complement repeats); **B**. The number and type of SSRs in *Scutellaria* cp genome

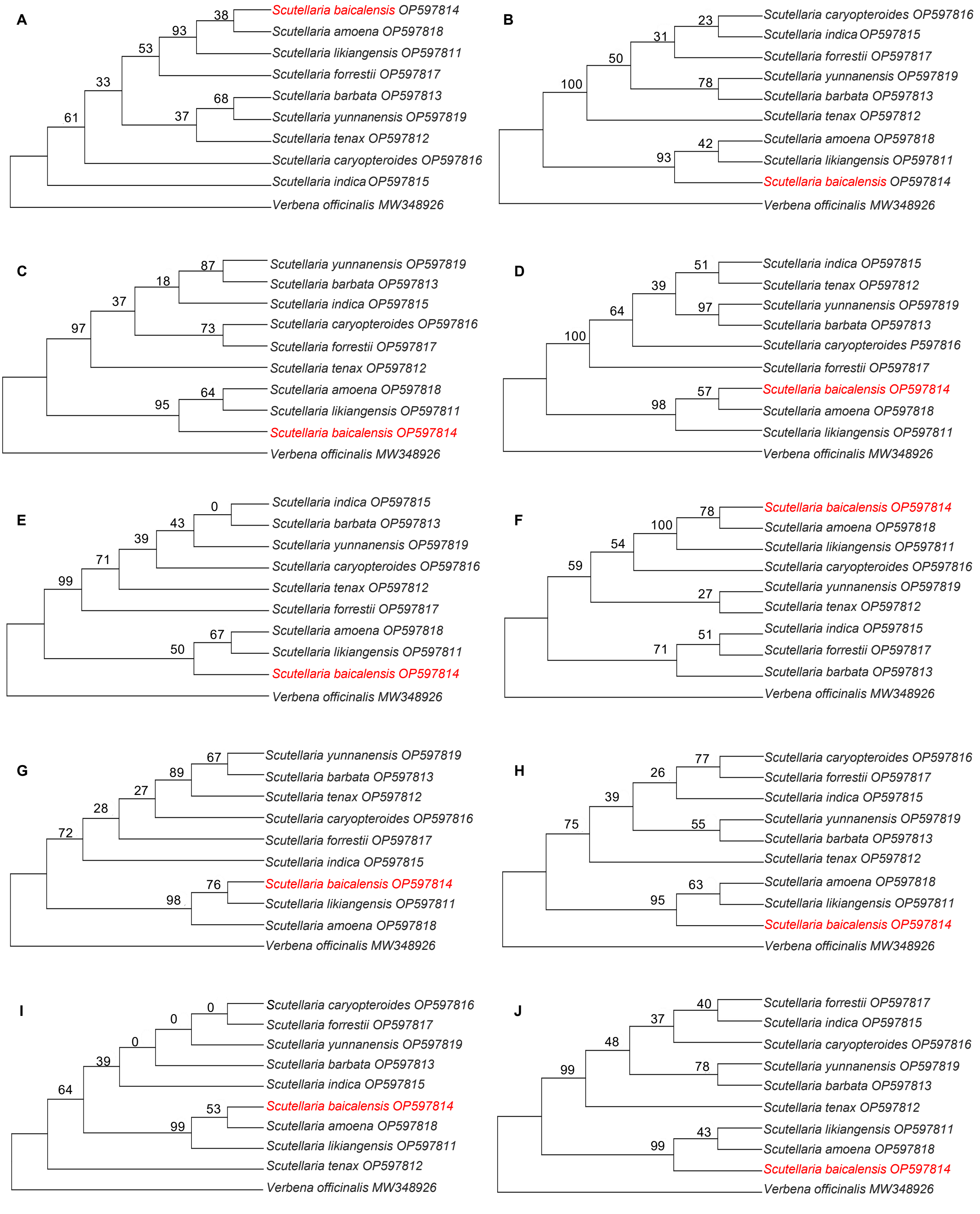

Fig. S4. Phylogenetic tree created using the ML technique based on the cp genome's several IGS (**A**. *accD-psaI*; **B**. *matK-rps16*; **C**. *ndhC-trnV-UAC*; **D**. *petN-psbM*; **E**. *psbE-petL*; **F**. *rbcL-accD*; **G**. *rpl16-rps3*; **H**. *rps16-trnQ-UUG*; **I**. *trnN-GUU-trnR-ACG*; **J**. four IGSs (*matK-rps16*, *ndhC-trnV-UAC*, *psbE-petL*, and *rps16-trnQ-UUG*))

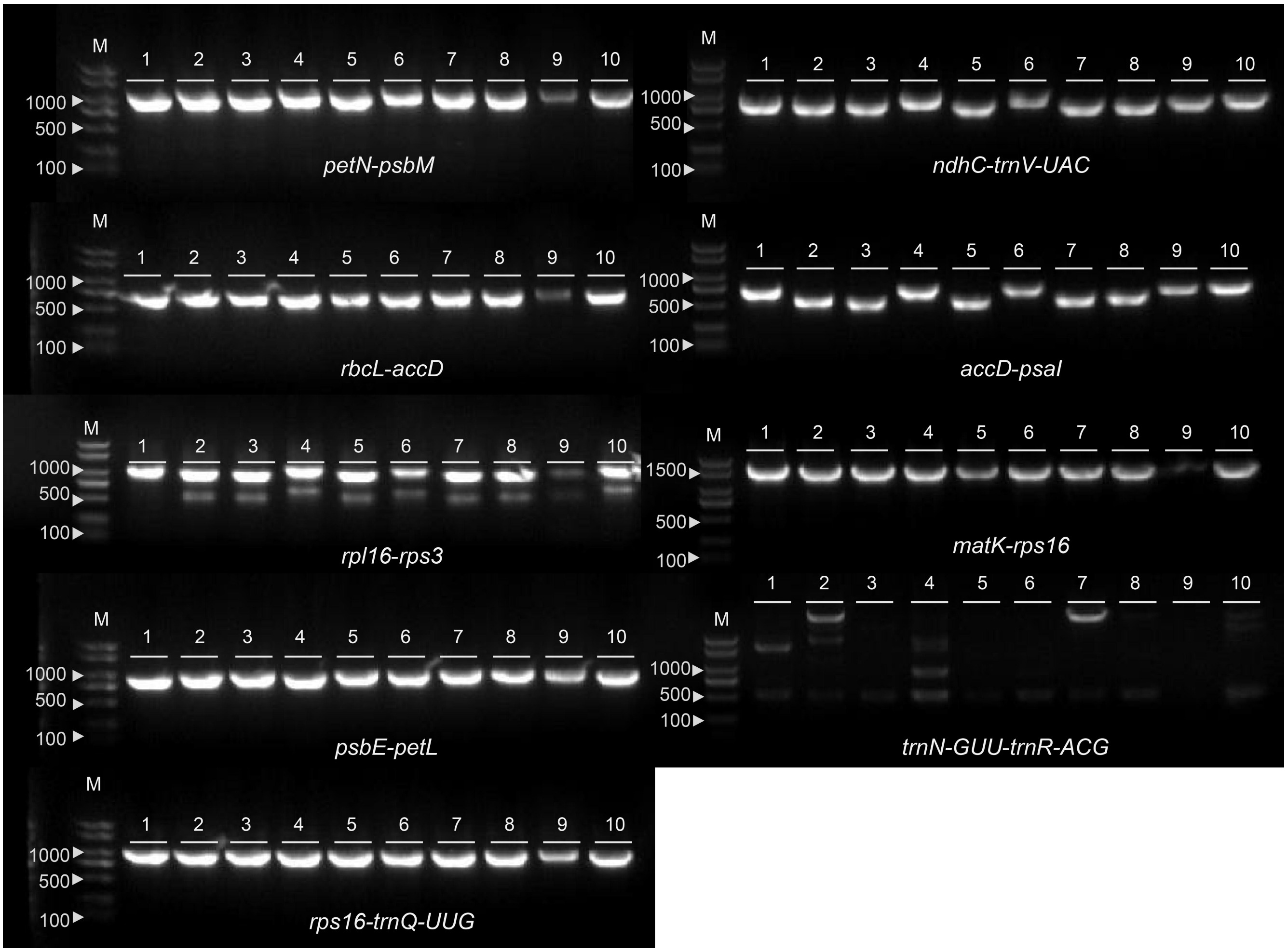

Fig. S5. The gel electrophoresis results of nine IGS PCR products. Lane M was the marker of DL2000 Plus. The lanes from left to right corresponded: S1. *S. likiangensis*; S2. *S. barbata*; S3. *S. yunnanensis*; S4. *S. amoena*; S5. *S. tenax*; S6. *S. baicalensis*; S10. *S. purpureocardia*; S11. *S. weishanensis*; S12. *S. teniana*; S13. *S. kingiana*.

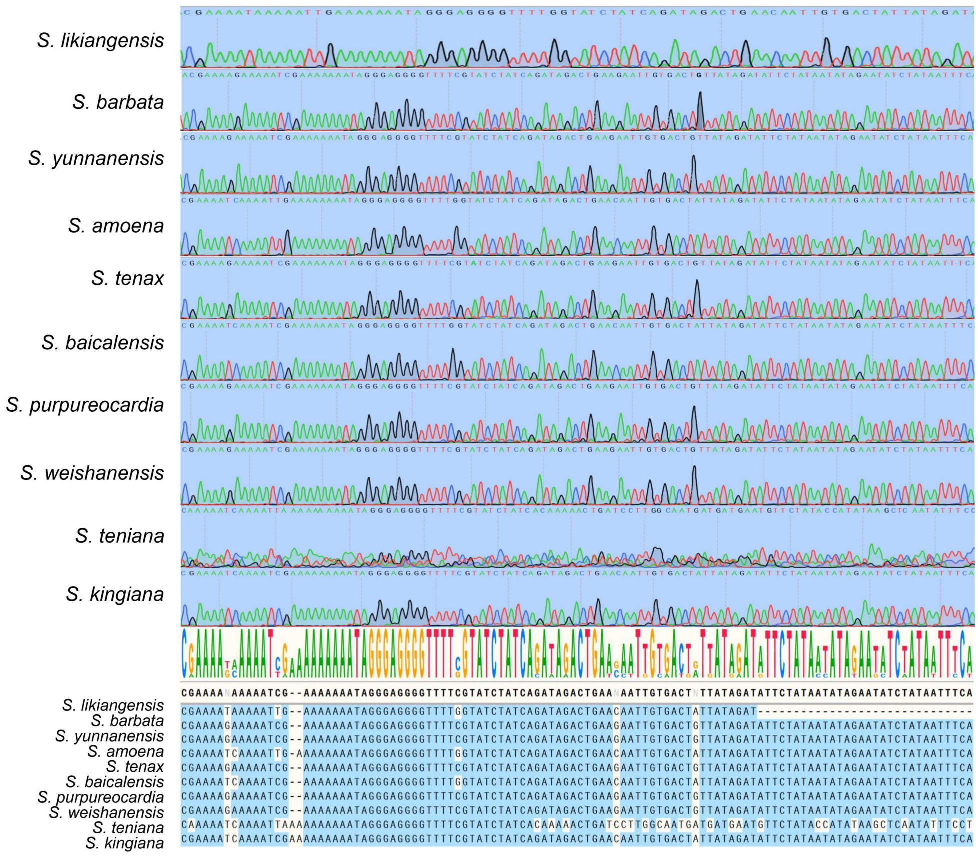

Fig. S6A. Sequencing chromatograms of the *rps16-trnQ-UUG* barcode in seven *Scutellaria* species.

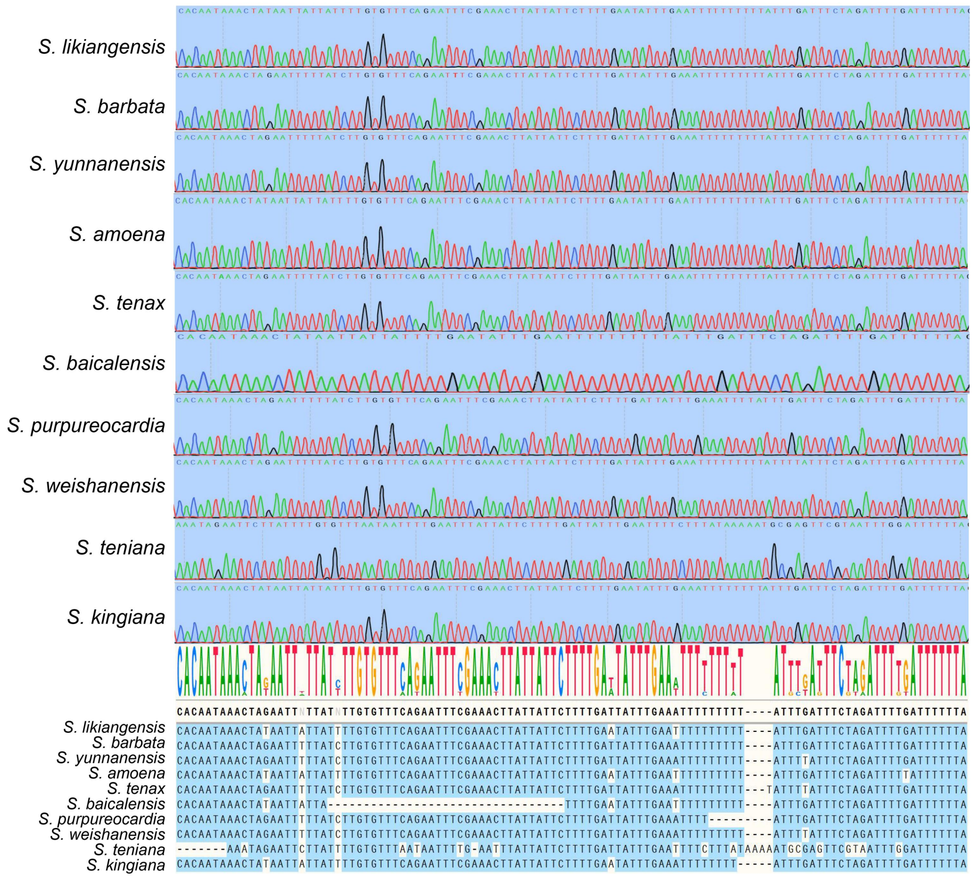

Fig. S6B. Sequencing chromatograms of the *ndhC-trnV-UAC* barcode in seven *Scutellaria* species.

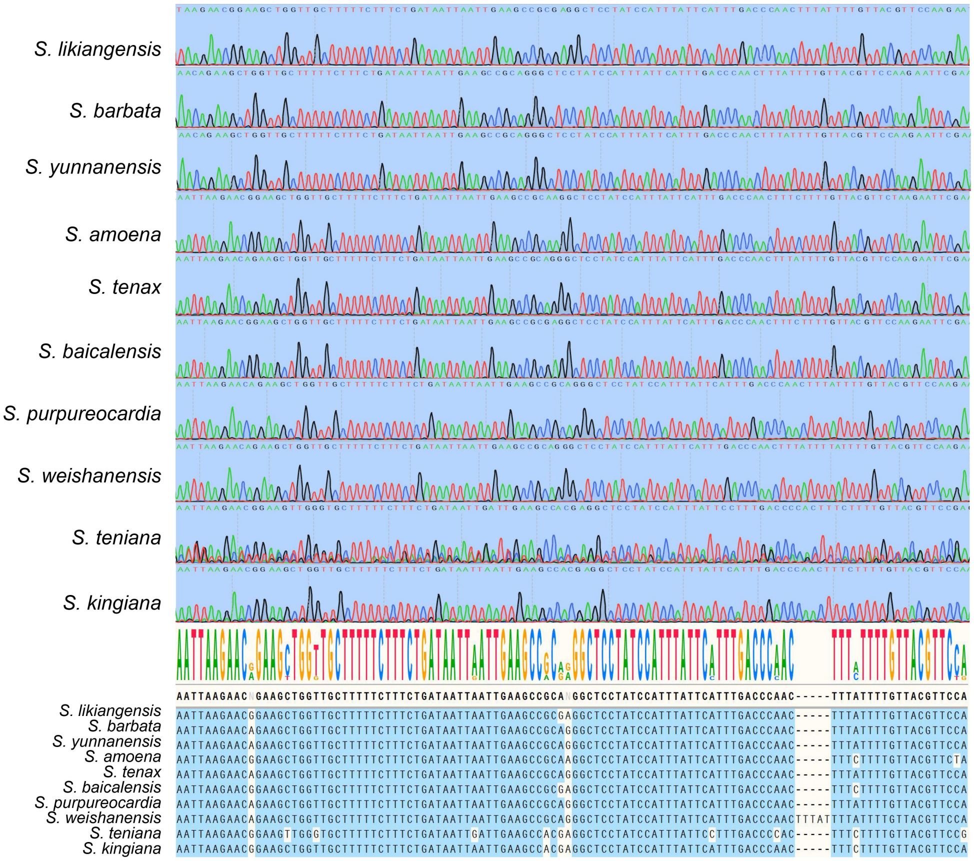

Fig. S6C. Sequencing chromatograms of the *matK-rps16* barcode in seven *Scutellaria* species.

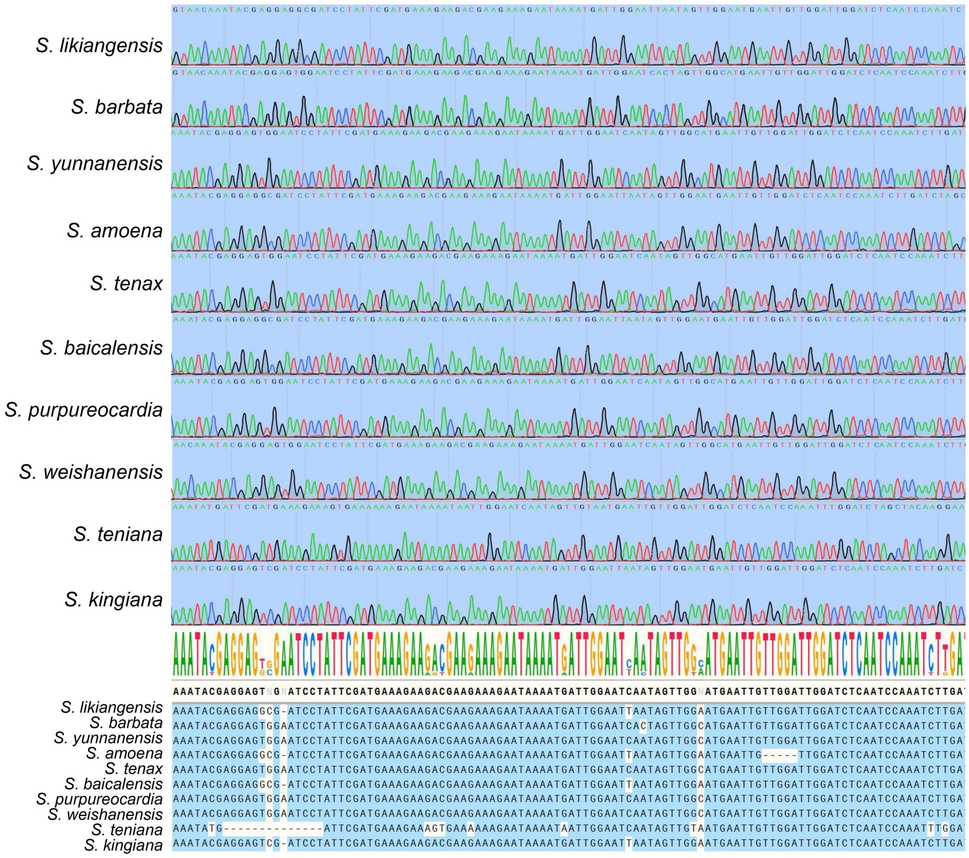

Fig. S6D. Sequencing chromatograms of the *psbE-petL* barcode in seven *Scutellaria* species.

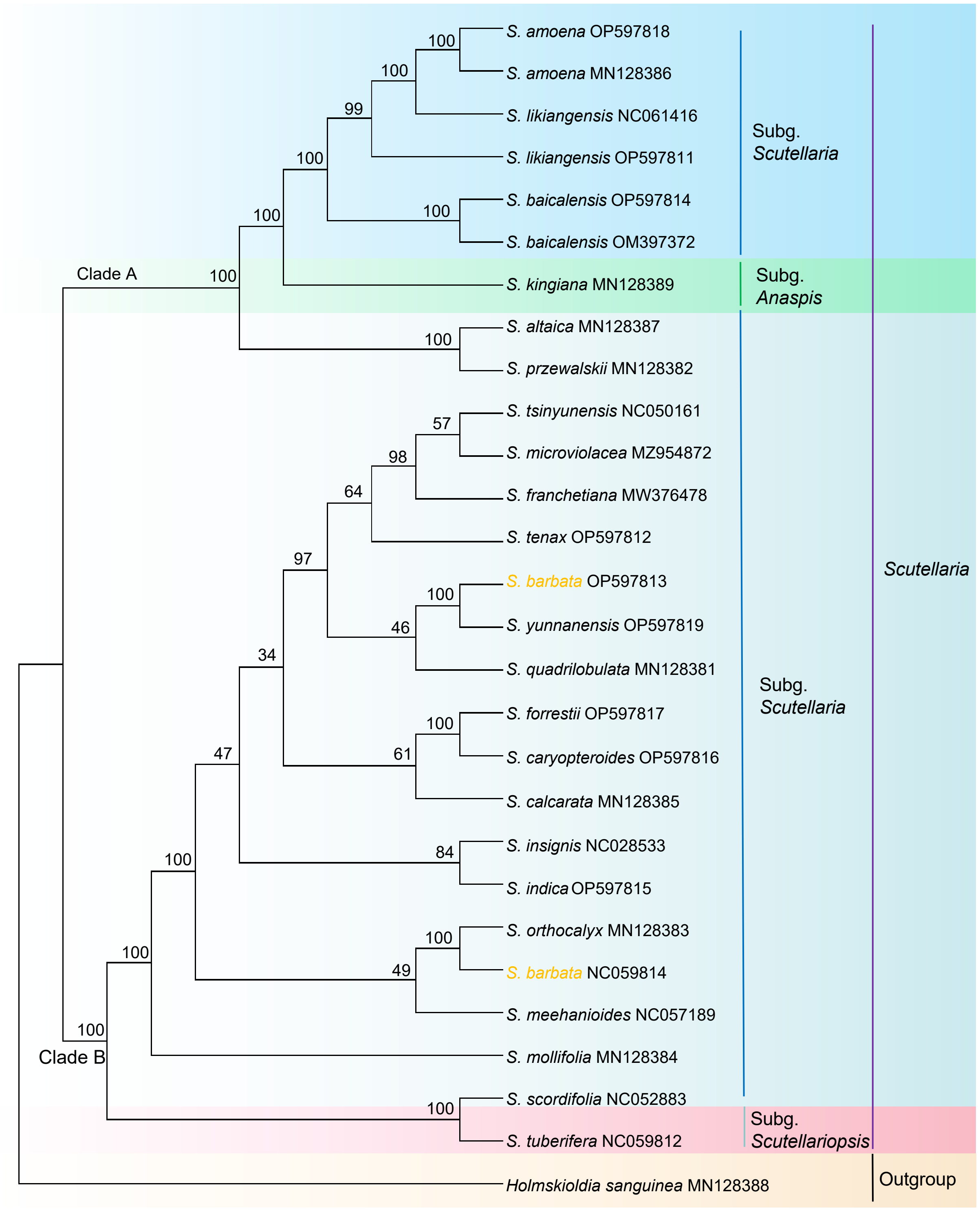

Fig. S7. The ML phylogenetic tree based on shared coding sequences (CDS) of the 24 species. The bootstrap support values are listed at each node.

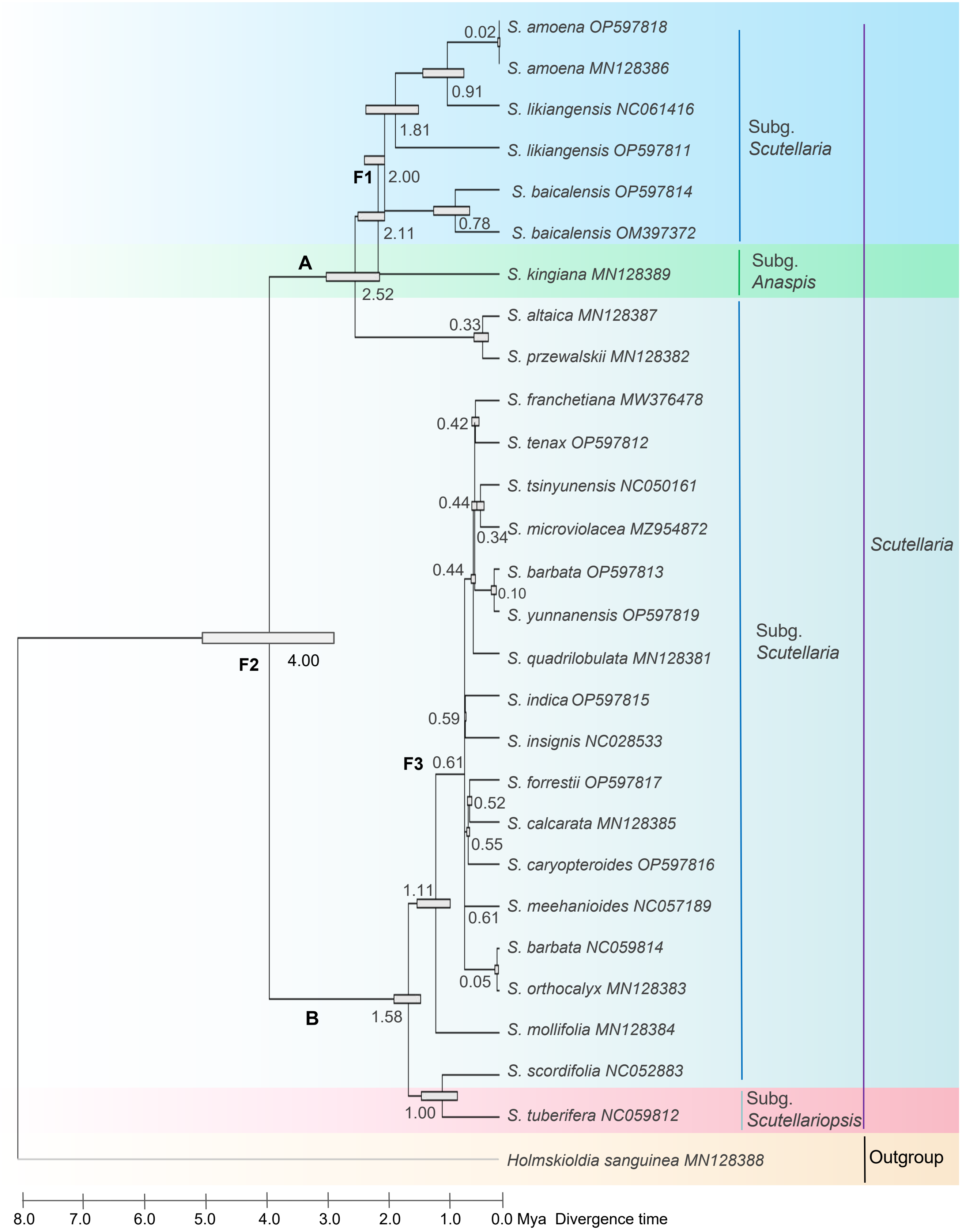

Fig. S8. Divergence times estimation based on cp genomes. The node ages are given for each node.
